# Comparative Analysis of Glycosidic Aroma Compound Profiling in Three *Vitis vinifera* Varieties by Using Ultra-High-Performance Liquid Chromatography Quadrupole-Time-of-Flight Mass Spectrometry

**DOI:** 10.3389/fpls.2021.694979

**Published:** 2021-06-24

**Authors:** Yi Wei, Zhuo Chen, Xin-Ke Zhang, Chang-Qing Duan, Qiu-Hong Pan

**Affiliations:** ^1^Center for Viticulture and Enology, College of Food Science and Nutritional Engineering, China Agricultural University, Beijing, China; ^2^Key Laboratory of Viticulture and Enology, Ministry of Agricultural and Rural Affairs, Beijing, China

**Keywords:** glycosidically bond aroma compound, quadrupole-time-of-flight mass spectrometry, grape berry, ripening stage, UHPLC

## Abstract

Glycosidic aroma compounds are the important precursors of volatile aroma in grapes, and they can be added with odorous aglycones *via* enzyme- or acid-catalyzed hydrolysis during wine fermentation and storage. Developing an analytical method for intact glycosides can provide the possibility to study the accumulation of these aroma precursors in grape berries. For this purpose, a Tandem Mass Spectrometry (MS/MS). database based on ultra-high-performance liquid chromatography quadrupole-time-of-flight mass spectrometry was built, covering multiple aglycone classes. Subsequently, the profiles of glycosidic aroma compounds in *Vitis vinifera* L. cv. Muscat Blanc, Riesling, and Chardonnay berries during maturation were investigated. Pentosyl-hexosides were the most abundant glycosides in all three varieties. Both composition and concentration of glycosidic aroma compounds varied obviously among grape varieties. Except for monoterpenol pentosyl-hexosides, most glycosides were kept almost stable in their concentrations during berry maturation. This research provides an approach to understand the variation of glycosidic aroma components from the perspective of aglycones and glycosides.

## Introduction

Different from free volatile aroma compounds, non-volatile glycosidic aroma compounds cannot be smelled; however, they are the important precursors of volatile aroma in grapes and wines. Glycosylation increases their water solubility and reduces reactivity, which in turn facilitates the transport, accumulation, storage, and even detoxification of volatiles in plants (Winterhalter and Skouroumounis, 1997; Sarry and Gunata, 2004; Hjelmeland and Ebeler, 2015). Most of the glycosides show higher concentrations than their free forms in mature grapes (Gunata et al., 1985b). During wine fermentation and storage, glycosides are conversed to free volatiles through enzymatic hydrolysis of glucosidases or acid hydrolysis (Gunata et al., 1985b; Parker et al., 2018). The glycoside is structurally composed of two moieties, namely, volatile aglycones and non-volatile glycones. Generally, the aglycones identified in grapes are terpenoids, norisoprenoids, straight-chain alcohols, volatile phenols, and benzenoids (Caffrey et al., 2020). Glycones are mainly disaccharide glycosides, followed by glucosides and trisaccharide glycosides. To date, the glucosides identified in grapes are β-d-glucopyranoside; the disaccharide glycosides are 6-*O*-α-l-rhamnopyranosyl-β-d-glucopyranoside, 6-*O*-α-l-arabinofuranosyl-β-d-glucopyranoside, and 6-*O*-α-l-apiofuranosyl-β-d-glucopyranoside (Gunata et al., 1985b; Voirin et al., 1990; Hjelmeland and Ebeler, 2015). In recent studies, the trisaccharide glycosides have been found in the form of hexosyl–pentosyl-hexoside, hexosyl–hexosyl-hexoside, and pentosyl–pentosyl-hexoside in grapes (Hjelmeland et al., 2015; Caffrey et al., 2020).

The accumulation patterns of glycosidic aroma compounds from different metabolic pathways show difference during the ripening in grapes. Most monoterpene glycosides increase significantly after veraison and reach to the top level in matured berries of Muscat grapes (Wilson et al., 1984; Gunata et al., 1985a). Norisoprenoids in glycosidic form are already present at a low level before veraison and increase throughout ripening in Muscat of Alexandria and Shiraz (Mathieu et al., 2005). To date, the glycosylation patterns of benzenoids are not very clear. The concentrations of both bound benzyl alcohol and bound 2-phenylethanol are relatively stable during the ripening (Fenoll et al., 2009). The concentrations of total glycosidic fatty acid derivatives maintain a relatively stable level and are much less than the sum of fatty acid-derived volatiles (Gao et al., 2016). However, due to the limitation of the detection methods (which will be mentioned in the following paragraph), most of these studies only analyze the volatile aglycones of glycosides, while the glycosylation patterns have not been clearly studied.

The most common method for the determination of glycosidic aroma compounds is to liberate the volatile aglycones by enzymatic or chemical hydrolysis and then determine them using gas chromatography-mass spectrometry (GC-MS). (Loscos et al., 2009; Metafa and Economou, 2013; Wang et al., 2020). However, this analysis method has some limitations. For monoterpenoids, the acid-catalyzed hydrolysis usually causes aglycone rearrangements, so that the compounds identified cannot represent the real structures of origin aglycones in samples (Skouroumounis and Sefton, 2000). By comparison, the acid-catalyzed hydrolysis may be more suitable for the analysis of norisoprenoid glycosides (Loscos et al., 2009). Additionally, the enzyme-catalyzed hydrolysis generally needs a long time for incubation, and the hydrolysis efficiency is directly interfered by the enzyme activity and the aglycone structures (Sarry and Gunata, 2004). In brief, the mode of hydrolysis could influence the separation of aglycones from glycosides. Early researchers reported a method of GC-MS pre-column derivatization with trimethylsilyl (TMS) and trifluoroacetyl (TFA) to analyze terpene glycosides without hydrolysis pretreatment (Voirin et al., 1992). But when the target compounds turn to multiple classes of aglycones, derivatization makes the complete separation of glycosides more complex and difficult.

To obtain intact information of glycones, the liquid chromatography mass spectrometry (LC-MS) was used (Nasi et al., 2008; Schievano et al., 2013). Due to the limited separation effect of chromatogram from LC-MS, the ultra-high-performance liquid chromatography mass spectrometry (UHPLC-MS) becomes a better choice, and the ultra-high-performance liquid chromatography quadrupole-time-of-flight mass spectrometry (UHPLC-Q-TOF-MS) has been applied to directly detect the intact glycosides without the risks of aglycone rearrangements. In most relevant studies, only a single class of volatile aglycones (mainly monoterpenes) was identified at a time using UHPLC-Q-TOF-MS (Flamini et al., 2014, 2018; Hjelmeland et al., 2015; Caffrey et al., 2019). A recent study demonstrated that the glycosides with multiple aglycones in Riesling could be simultaneously separated using the porous graphitic carbon (PGC) column (Caffrey et al., 2020). An ACE C18-PFP column was also demonstrated in order to separate the multiple types of glycosidic aroma precursors in Muscat of Alexandria (Cebrian-Tarancon et al., 2021). However, most of these previous studies were related to limited classes of volatile aglycones or ripening stages of berries, which may not be conductive to the comprehensive study on the profiles of glycosidic aroma compounds during grape maturation.

This study aimed to establish a UHPLC-Q-TOF-MS method for the simultaneous analysis of multiple classes of glycosidic aroma compounds and to help us understand the profiling of glycosidic aroma precursors in grape berries. To this end, a database was established in conjunction with GC-MS, including 60 glycosidic aroma compounds from berries of 10 grape varieties, covering multiple aglycone classes, which lays a foundation for the UHPLC-Q-TOF-MS identification. Then, we evaluated the variation of glycosidic aroma compounds in three grape varieties during the ripening.

## Materials and Methods

### Chemicals

Pure standards of aroma compounds, namely, polyvinylpolypyrrolidone (PVPP) and 4-methyl-2-pentanol methanol, were purchased from Sigma-Aldrich (St. Louis, MO, USA). β-d-glucolactone and *n*-octyl-β-d-glucopyranoside were purchased from Sangon Biotech (Shanghai, China). Rapidase® AR2000 commercial preparation with glycosidase side activities was purchased from DSM Oenology (Delft, The Netherlands). Methanol, dichloromethane, citric acid, and sodium phosphate dibasic dodecahydrate (analytical grade) were purchased from Beijing Chemical Works (Beijing, China). LC-MS grade acetonitrile, formic acid, and methanol were purchased from Honeywell (Morristown, NJ, USA).

### Plant Materials

Ten *V. vinifera* varieties were used to establish a reference database, the objective of which was to understand the aglycone composition of aroma glycosides and their MS/MS information. The grape varieties included Petit Manseng (marked as MAN), Aranèle (marked as ARA), Viognier (marked as VIO), Italian Riesling (marked as ITAR), Sauvignon Blanc (marked as SAUB), Roussanne (marked as ROU), Gewürztraminer (marked as GWU), Muscat Blanc, Riesling, and Chardonnay. The grape berries were harvested from the Shangzhuang Experimental Station of China Agricultural University (Beijing, China) at the ripening stage.

For spanning the variation of grape berry maturation, the grape samples were harvested in 2018 from the same experimental station at four different maturities according to E-L stage (Coombe, 1995). Three *V. vinifera* varieties (two clones per variety) were harvested at E-L 34, E-L 35, E-L 36, and E-L 37, which represented the stages before, during, and after veraison (Coombe, 1995): Muscat Blanc (clones 455 and 826, both grafted to 5BB rootstock, marked as M1 and M2), Riesling (clone C49 own rooted and C49 grafted to 1103P rootstock, marked as R1 and R2), and Chardonnay (clone 76 grafted to 1103P rootstock and clone 277 grafted to 5BB rootstock, marked as C1 and C2).

The vines were arranged in north–south oriented rows spaced 2.5 m apart with a distance of 1.2 m between two vines in each row. At each sampling time point, two or three clusters were harvested from each side of vines, at least 60 berries were randomly collected from the top, middle, and bottom of each cluster. The duplicate samples were harvested from at least three different vines. Grapes were immediately flash frozen in liquid nitrogen after harvest and kept at −70°C until extraction.

### Sample Preparation

The samples were prepared based on the reported method developed by our group (Wang et al., 2020). Approximately, 50 g of whole grape berries, with pedicels and seeds removed, were blended with 1 g PVPP and 0.5 g β-d-glucolactone and ground into powder in liquid nitrogen using a basic grinder (IKA, Staufen, Germany). The flesh was immediately centrifuged at 8,000 rpm for 10 min after maceration at 4°C for 4 h. The supernatant was collected for extraction of glycosidic aroma compounds. The Cleanert PEP-SPE cartridge (150 mg/6 mL, Bonna-Agela Technologies, China) was conditioned by 10 mL of methanol and 10 mL of water separately before 2 mL of clear juice was added. Then the cartridge was washed with 2 mL of water and 5 mL of dichloromethane to remove water-soluble compounds and free volatiles, respectively. The glycosidic aroma compounds were eluted in 20 mL of methanol.

### Gas Chromatography-Mass Spectrometry Analysis

The eluate described above was concentrated to dryness by a rotary evaporator under vacuum at 30°C and was redissolved in 10 mL of citrate/phosphate buffer. The buffer was divided evenly and was transferred to a falcon tube, respectively. The AR2000 solution (100 μL, 100 g/L) was added to each tube, and the sample was vortexed (Wang et al., 2020). The sample was sealed and placed in an incubator at 37°C for 16 h. The enzymatic hydrolysate (5 mL) with 1 g of NaCl and 10 μL of 4-methyl-2-pentanol (1 g/L, internal standard) was blended in a sample vial and tightly capped with a PTFE-silicone septum. According to our previous report, the aroma compounds were concentrated by headspace solid-phase micro-extraction (Lan et al., 2019).

The analyses were performed on an Agilent 6890 GC coupled to an Agilent 5975 MS, fitted with a 60 m × 0.25 mm × 0.25 μm HP-INNOWAX capillary column (J&W Scientific, Folsom, CA, USA) according to a method reported previously (Lan et al., 2019). Helium (>99.999%) was used as the carrier gas at 1 mL/min. The inlet was set in the splitless mode. The operating conditions were as follows: injector, 250°C; ion source, 230°C; and interface, 250°C. The temperature program was performed by maintaining temperature at 50°C for 1 min, heating up to 220°C at 3°C/min, and holding at 220°C for 5 min. Retention indices (RIs) were calculated after analyzing a C_6_-C_24_ *n*-alkane series (Supelco, Bellefonte, PA, USA) under the same chromatographic conditions. The aroma compounds were identified based on the RIs and the mass spectra matching in the standard NIST11 MS database.

### Ultra-High-Performance Liquid Chromatography Quadrupole-Time-of-Flight Mass Spectrometry Analysis

The sample preparation for the UHPLC-Q-TOF-MS analysis was the same as the GC-MS. The Cleanert PEP-SPE cartridge (500 mg/6 mL, Bonna-Agela Technologies, China) was used. Twenty milliliters of methanol eluate described above was concentrated to dryness by a rotary evaporator under vacuum at 30°C and was redissolved in 2 mL methanol. Of note, 10 μL of *n*-octyl-β-d-glucopyranoside (100 mg/L, internal standard) was added with 1 mL of extracting solution. The sample was vortexed and filtered by 0.22-μm organic phase membrane filters (Jinteng Experimental Equipment Co., Ltd, Tianjin, China). All the analyses were performed in duplicate.

The analyses were performed on an Agilent 1290 Infinity II UHPLC in tandem with an Agilent 6545 Q-TOF-MS. The sample (5 μL) was injected into a Zorbax reversed-phase column (RRHD SB-C18 3 × 150 mm, 1.7 μm) and analyzed in the negative mode using an Agilent Dual ESI Jet Steam source. The column was heated at 35°C. The mobile phases were 0.1% (v/v) acetic acid in water (A) and 0.1% (v/v) acetic acid in acetonitrile (B) with a flow rate of 0.4 mL/min. The elution program was consisted of a linear gradient from 5% B to 45% B over 22 min and a linear gradient to 100% B over 5 min, ending with a 10 min hold at 100% B.

According to a previously described method (Flamini et al., 2014; Godshaw et al., 2019), a scan range of mass-to-charge ratio (*m/z*) 100–1,050 was utilized for the MS mode and *m/z* 20–800 for the auto MS/MS mode. The collection frequency was 4 spectra/s. The nozzle voltage was set to 1,000 V, capillary voltage to 3,500 V, fragmentor voltage to 175 V, sheath gas (nitrogen) at 10 L/min at 400°C, drying gas at 8 L/min at 350°C, and the nebulizer to 35 psig. A standard mixture G1969-8500 (Supelco, Inc., Bellefonte, PA, USA) was used to ensure accurate mass calibration. The mass deviation was limited with ±0.2 ppm. Reference ions were TFANH4 (*m/z* 112.9856) and HP-0921 (*m/z* 1033.9881) using an API-TOF Reference Mix (Agilent, part no. G1969-85001). The compounds were fragmented with collision energies of 15, 20, and 30 eV.

The compounds bearing hydroxyl group identified by the GC-MS in 10 grape varieties were screened out as candidate aglycones. Putative glycosidic aroma compounds were added into the Agilent MassHunter Personal Compound Database Library (PCDL) Manager to create a personal molecular formula database based on those aglycones above and previously identified glycones in grapevine (Gunata et al., 1985b; Voirin et al., 1990; Mateo and Jimenez, 2000; Hjelmeland et al., 2015). The data were searched for matching the PCDL formula database (score ≥80 and mass error ≤ 5ppm) using the Find-by-Auto MS/MS and Identify Compounds algorithm and then added into the PCDL structural formula database; the data matching against the PCDL formula database were analyzed by Molecular Formula Generation (MFG) to generate the molecular formula, and the potential compounds with typical glycoside fragments were added into the PCDL structural formula database too. The molecular structures of compounds were speculated by comparing MS/MS spectra with the PCDL structural formula database and online database (ChemSpider) using the Molecular Structure Correlator (MSC). The data of compounds with reasonable fragmentation pattern and other compounds previously identified (Guth, 1997; Flamini et al., 2014; Ghaste et al., 2015; Yang et al., 2019) were extracted to update the database of potential matches with corresponding retention times. The precursor ion was used as the quantifier. The peak was specified with a criterion of signal/noise ratio of 10. The semiquantitative data were calculated by the ratio of the peak area of the compound to the peak area of the internal standard within each analysis. All the semiquantitative data of all compounds were manually integrated using MassHunter software (Version 8.00, Agilent technology).

### Statistical Analysis

All analysis assessed differences using a *p*-value of 0.05. A one-way ANOVA was performed to test the differences among treatments by the Duncan's multiple range test in RStudio (v1.2.5019, Boston, MA, USA). To visualize the differences, the principal component analysis (PCA) was carried out by using SIMCA 14.1 (Umetrics, Malmö, Sweden), and heatmap was carried out by using MataboAnalyst 4.0 (https://www.metaboanalyst.ca/) (Chong et al., 2019). In the data matrix for multivariate analysis, the concentrations of all compounds for the three varieties from E-L 34 to E-L 37 were used, in which two biological replicates and two analytical replicates for each sample were used for the PCA analysis, and their mean values were used for heatmap. “Auto scaling” was used for heatmap data scaling.

## Results and Discussion

### A MS/MS Spectrum Database Establishment of Glycosidic Aroma Compounds

Using GC-MS, we identified 51 aglycones of glycosidic aroma compounds from the mature berries of 10 grape varieties (Supplementary Table 1). Hexose, pentose, and rhamnose were assigned to bind to the aglycones above. Molecular formulas and exact neutral masses were added into the database using MassHunter PCDL Manager. Some other variants of glycosidic aroma compounds reported in the literature of *V. vinifera* were also supplemented into the database (Guth, 1997; Mateo and Jimenez, 2000; Fernandez-Gonzalez and Di Stefano, 2004; Flamini et al., 2014; Ghaste et al., 2015). The database was used for the qualitative analysis by UHPLC-Q-TOF-MS. All tentative compounds exhibited a <5 ppm error between the exact mass and the theoretical mass.

**Figure 1:**
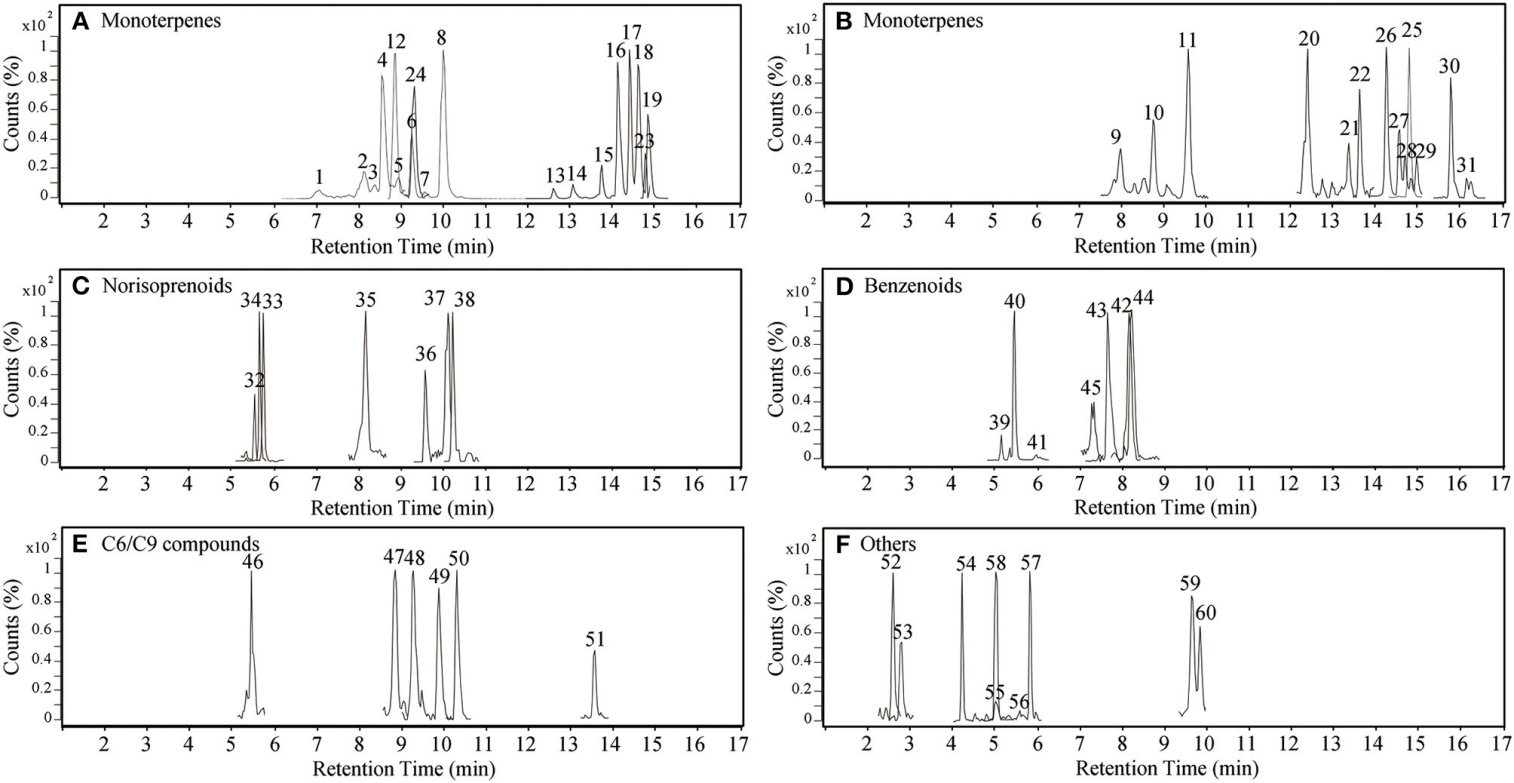
Selected extracted ion chromatograms of glycosidic aroma compounds in grape berries from 10 varieties. Separate aglycone classes in each panel are: **(A,B)** monoterpenes, **(C)** norisoprenoids, **(D)** benzenoids, **(E)** C6/C9 compounds, and **(F)** others. The peak numbers correspond to compounds mentioned in Table 1, Supplementary Tables 2, 3, Figures 5, 6.

Using UHPLC-Q-TOF-MS, 60 compounds were tentatively identified as glycosidic aroma compounds in 10 grape varieties (Table 1, Supplementary Table 2). The extracted ion chromatograms of all these compounds were shown in Figures 1A–F. These compounds were divided into five groups based on the classes of aglycone: monoterpenes, norisoprenoids, benzenoids, C6/C9 compounds, and others. Some of the glycosides have been reported previously (Flamini et al., 2014; Hjelmeland et al., 2015; Godshaw et al., 2019; Cebrian-Tarancon et al., 2021). It was emphasized that 2 new types of monoterpene glycosides and other 15 types of glycosides were putatively identified in grape berries for the first time. Structural identification of each new compound is further described below.

**Table 1:** Primary mass spectrum information of glycosidic aroma compounds identified in 10 grape varieties.

**No.^**a**^**	**Retention time (min)**	**Compound**	**Formula**	**Exact mass (MW)**	**Precursor ion**	**Quantifier (*m/z*)**	**MS/MS sugar or aglycone loss**	**Aglycone fragments**	**CE (eV)**
**Monoterpenes**									
1	7.21	Monoterpene-diol pentosyl-hexoside-1	C_21_H_36_O_11_	464.2258	[M + COOH]^−^	509.2241	293.0874, 331.1762	nf^*b*^	20
2	8.17	Monoterpene-diol pentosyl-hexoside-2	C_21_H_36_O_11_	464.2258	[M + COOH]^−^	509.2241	293.0874, 331.1762	nf	20
3	8.43	Monoterpene-diol pentosyl-hexoside-3	C_21_H_36_O_11_	464.2258	[M + COOH]^−^	509.2241	293.0874, 331.1762	nf	20
4	8.70	Monoterpene-diol pentosyl-hexoside-4	C_21_H_36_O_11_	464.2258	[M + COOH]^−^	509.2241	293.0874, 331.1762	nf	20
5	8.97	Monoterpene-diol pentosyl-hexoside-5	C_21_H_36_O_11_	464.2258	[M + COOH]^−^	509.2241	293.0874, 331.1762	nf	20
6	9.35	Monoterpene-diol pentosyl-hexoside-6	C_21_H_36_O_11_	464.2258	[M + COOH]^−^	509.2241	293.0874, 331.1762	nf	20
7	9.63	Monoterpene-diol pentosyl-hexoside-7	C_21_H_36_O_11_	464.2258	[M + COOH]^−^	509.2241	293.0874, 331.1762	nf	20
8	10.11	Monoterpene-diol pentosyl-hexoside-8	C_21_H_36_O_11_	464.2258	[M + COOH]^−^	509.2241	293.0874, 331.1762	nf	20
9	8.13	Monoterpene-diol hexosyl-pentosyl-hexoside-1	C_27_H_46_O_16_	626.2786	[M + COOH]^−^	671.2767	331.1760, 463.2182	nf	30
10	8.83	Monoterpene-diol hexosyl-pentosyl-hexoside-2	C_27_H_46_O_16_	626.2786	[M + COOH]^−^	671.2767	331.1760, 463.2182	nf	30
11	9.66	Monoterpene-diol hexosyl-pentosyl-hexoside-3	C_27_H_46_O_16_	626.2786	[M + COOH]^−^	671.2767	331.1760, 463.2182	nf	30
12	8.94	Dihydro-monoterpene-diol pentosyl-hexoside	C_21_H_38_O_11_	466.2414	[M + COOH]^−^	511.2397	149.0454, 465.2341	nf	20
13	12.61	Monoterpenol pentosyl-hexoside-1	C_21_H_36_O_10_	448.2308	[M + COOH]^−^	493.2242	293.0875, 315.1806	nf	20
14	13.13	Monoterpenol pentosyl-hexoside-2	C_21_H_36_O_10_	448.2308	[M + COOH]^−^	493.2242	293.0875, 315.1806	nf	20
15	13.77	Monoterpenol pentosyl-hexoside-3	C_21_H_36_O_10_	448.2308	[M + COOH]^−^	493.2242	293.0875, 315.1806	nf	20
16	14.20	Monoterpenol pentosyl-hexoside-4	C_21_H_36_O_10_	448.2308	[M + COOH]^−^	493.2242	293.0875, 315.1806	nf	20
17	14.48	Monoterpenol pentosyl-hexoside-5	C_21_H_36_O_10_	448.2308	[M + COOH]^−^	493.2242	293.0875, 315.1806	nf	20
18	14.64	Monoterpenol pentosyl-hexoside-6	C_21_H_36_O_10_	448.2308	[M + COOH]^−^	493.2242	293.0875, 315.1806	nf	20
19	14.84	Monoterpenol pentosyl-hexoside-7	C_21_H_36_O_10_	448.2308	[M + COOH]^−^	493.2242	293.0875, 315.1806	nf	20
20	12.46	Monoterpenol hexosyl-pentosyl-hexoside-1	C_27_H_46_O_15_	610.2837	[M + COOH]^−^	655.2818	315.1806, 447.2223	nf	20
21	13.47	Monoterpenol hexosyl-pentosyl-hexoside-2	C_27_H_46_O_15_	610.2837	[M + COOH]^−^	655.2818	315.1806, 447.2223	nf	20
22	13.73	Monoterpenol hexosyl-pentosyl-hexoside-3	C_27_H_46_O_15_	610.2837	[M + COOH]^−^	655.2818	315.1806, 447.2223	nf	20
23	14.84	Monoterpenol rhamnosyl-hexoside	C_22_H_38_O_10_	462.2465	[M + COOH]^−^	507.2446	163.0612, 205.0717	nf	20
24	9.41	Geranic acid hexosyl-hexoside	C_22_H_36_O_12_	492.2207	[M + COOH]^−^	537.2195	329.1599	nf	20
25	14.93	Geranic acid rhamnosyl-hexoside	C_22_H_36_O_11_	476.2258	[M + COOH]^−^	521.2245	163.0612, 307.1034	167.1075	20
26	14.37	Geranic acid pentosyl-hexoside-1	C_21_H_34_O_11_	462.2101	[M + COOH]^−^	507.2088	293.0875	167.1075	20
27	14.63	Geranic acid pentosyl-hexoside-2	C_21_H_34_O_11_	462.2101	[M + COOH]^−^	507.2088	293.0875	167.1075	20
28	14.73	Geranic acid pentosyl-hexoside-3	C_21_H_34_O_11_	462.2101	[M + COOH]^−^	507.2088	293.0875	167.1075	20
29	15.11	Geranic acid pentosyl-hexoside-4	C_21_H_34_O_11_	462.2101	[M + COOH]^−^	507.2088	293.0875	167.1075	20
30	15.82	Citronellol pentosyl-hexoside-1	C_21_H_38_O_10_	450.2465	[M + COOH]^−^	495.2451	317.1961	nf	20
31	16.23	Citronellol pentosyl-hexoside-2	C_21_H_38_O_10_	450.2465	[M + COOH]^−^	495.2451	317.1961	nf	20
**Norisoprenoids**									
32	5.74	Vomifoliol hexoside-1	C_19_H_30_O_8_	386.1941	[M + COOH]^−^	431.1930	179.0554	205.1231	20
33	5.89	Vomifoliol hexoside-2	C_19_H_30_O_8_	386.1941	[M + COOH]^−^	431.1930	179.0554	205.1231	20
34	5.80	Vomifoliol pentosyl-hexoside	C_24_H_38_O_12_	518.2363	[M – H]^−^	517.2288	293.0877	205.1229	20
35	8.36	Vomifoliol rhamnosyl-hexoside	C_25_H_40_O_12_	532.2520	[M + COOH]^−^	577.2499	307.1044, 325.1126	nf	20
36	9.70	3-Oxo-α-ionol/3-hydroxy-β-damascenone pentosyl-hexoside-1	C_24_H_38_O_11_	502.2414	[M + COOH]^−^	547.2396	311.0984	nf	20
37	10.30	3-Oxo-α-ionol/3-hydroxy-β-damascenone pentosyl-hexoside-2	C_24_H_38_O_11_	502.2414	[M + COOH]^−^	547.2396	311.0984	nf	20
38	10.36	3-Oxo-α-ionol/3-hydroxy-β-damascenone rhamnosyl-hexoside	C_25_H_40_O_11_	516.2571	[M + COOH]^−^	561.2547	307.1044, 325.1126	nf	20
**Benzenoids**									
39	5.33	Benzyl alcohol pentosyl-hexoside-1	C_18_H_26_O_10_	402.1526	[M – H]^−^	401.1457	269.1024, 293.0871	nf	20
40	5.54	Benzyl alcohol pentosyl-hexoside-2	C_18_H_26_O_10_	402.1526	[M – H]^−^	401.1457	269.1024, 293.0871	nf	20
41	6.13	Benzyl alcohol pentosyl-hexoside-3	C_18_H_26_O_10_	402.1526	[M – H]^−^	401.1457	269.1024, 293.0871	nf	20
42	8.44	Benzyl alcohol hexoside	C_13_H_18_O_6_	270.1103	[2M – H]^−^	539.2106	101.0244	177.0558	15
43	7.87	β-Phenylethanol pentosyl-hexoside	C_19_H_28_O_10_	416.1682	[M – H]^−^	415.1600	283.1168	nf	15
44	8.50	β-Phenylethanol rhamnosyl-hexoside	C_20_H_30_O_10_	430.1839	[M + COOH]^−^	475.1823	325.1119	nf	15
45	7.51	Methyl-salicylate rhamnosyl-hexoside	C_20_H_28_O_12_	460.1581	[M + COOH]^−^	505.1568	307.1049	151.0402	20
**C6/C9 compounds**									
46	5.62	3-Hexen-1-ol hexosyl-hexoside	C_18_H_32_O_11_	424.1945	[M – H]^−^	423.1866	261.1340	nf	20
47	9.03	1-Nonanol pentosyl-hexoside	C_20_H_38_O_10_	438.2465	[M + COOH – H2O]^−^	465.2332	293.0870	nf	15
48	9.36	1-Hexanol pentosyl-hexoside-1	C_17_H_32_O_10_	396.1995	[M + COOH]^−^	441.1978	263.1496	nf	20
49	9.90	1-Hexanol pentosyl-hexoside-2	C_17_H_32_O_10_	396.1995	[M + COOH]^−^	441.1978	263.1496	nf	20
50	10.35	1-Hexanol rhamnosyl-hexoside	C_18_H_34_O_10_	410.2152	[M + COOH]^−^	455.2143	263.1496	nf	20
51	13.57	3-Hexen-1-ol hexosyl-pentosyl-hexoside	C_23_H_40_O_15_	556.2367	[M – H – H2O]^−^	537.2195	323.0967, 221.0658	nf	20
**Others**									
52	2.72	Isopropyl-alcohol pentosyl-hexoside	C_14_H_26_O_10_	354.1526	[M – H]^−^	353.1459	221.1026	nf	20
53	2.93	Furaneol pentosyl-hexoside	C_17_H_26_O_12_	422.1424	[M – H]^−^	421.1353	289.0919	nf	20
54	4.34	2-Butanol pentosyl-hexoside	C_15_H_28_O_10_	368.1682	[M + COOH]^−^	413.1671	235.1182	nf	20
55	5.21	3-Methyl-1-butano pentosyl-hexoside-1	C_16_H_30_O_10_	382.1839	[M – H]^−^	381.1771	249.1345	nf	20
56	5.70	3-Methyl-1-butano pentosyl-hexoside-2	C_16_H_30_O_10_	382.1839	[M – H]^−^	381.1771	249.1345	nf	20
57	5.97	3-Methyl-1-butano pentosyl-hexoside-3	C_16_H_30_O_10_	382.1839	[M – H]^−^	381.1771	249.1345	nf	20
58	5.18	3-Methyl-2-buten-1-ol pentosyl-hexoside	C_16_H_28_O_10_	380.1682	[M – H]^−^	379.1593	249.1345	nf	15
59	9.76	1,10-Decanediol pentosyl-hexoside-1	C_21_H_40_O_11_	468.2571	[M + COOH]^−^	513.2550	335.2069	nf	20
60	9.98	1,10-Decanediol pentosyl-hexoside-2	C_21_H_40_O_11_	468.2571	[M + COOH]^−^	513.2550	335.2069	nf	20

a *The number (No.) corresponds to compounds mentioned in Supplementary Tables 2, 3, Figures 1, 5, 6.*

b *nf: no identifiable aglycone peaks were found in the MS/MS spectrum*.

In this study, the formate adduct ion [M + COOH]^−^ was more abundant than the deprotonated ion [M – H]^−^, mainly owing to the use of 0.1% formic acid in the mobile phase (Table 1). In most cases, a fragment of *m/z* 44.9988 corresponding to [COOH]^−^ was assigned to the precursor ion in negative ionization mode. As for glycone moieties, the typical fragment ions indicating the presence of hexose sugars had [Hex – H]^−^ at *m/z* 179.0573 and its consecutive fragment loss to produce *m/z* 161.0453, *m/z* 101.0245, *m/z* 89.0243, and *m/z* 59.0145 (Supplementary Table 2); the typical fragment ions of pentose sugars were *m/z* 149.0455 and *m/z* 131.0351, which were generated from the loss of one proton and its subsequent dehydration; the typical fragment ions of a rhamnose sugar was [Rhm – H]^−^ with *m/z* 163.0612 (Verardo et al., 2009; Caffrey et al., 2020; Cebrian-Tarancon et al., 2021). These fragment ions are not mentioned in Table 1 because the fragmentation patterns of sugar rings were repetitive. The *m/z* values for the loss of sugars or aglycones and aglycone fragments, which provided the information of the tentative structures of the glycosides, are included in Table 1.

#### Monoterpene Glycosides

Thirty-one monoterpene glycosides were tentatively confirmed. Monoterpenols have the same molecular formula C_10_H_18_O (MW = 154 Da), and they had a same fragment ion pattern under the same collision energies; similarly, monoterpene-diols also have the same molecular formula C_10_H_18_O_2_ (MW = 170 Da) and fragmentation information. So, it is difficult to accurately characterize the isomers of the monoterpene compounds. The typical fragmentation patterns of pentosyl-hexosides of monoterpenol, monoterpene-diol, and citronellol have been documented in relevant literatures (Schievano et al., 2013; Flamini et al., 2014; Caffrey et al., 2020; Cebrian-Tarancon et al., 2021). In this study, these typical ions like the pentosyl–hexosyl moiety (*m/z* 293.0874) were also detected (Table 1, Supplementary Table 2).

Two new types of monoterpene glycosides were characterized. Geranic acid rhamnosyl-hexoside was tentatively identified based on the precursor ions of [M + COOH]^−^ (*m/z* 521.2245) and [M – H]^−^ (*m/z* 475.2168), together with [Rhm + Hex – H – H_2_O]^−^ (*m*/*z* 307.1034), [Agl – H]^−^ (*m/z* 167.1075), and [Rhm – H]^−^ (*m*/*z* 163.0612) (Supplementary Table 2), which indicated the loss of aglycones, fragments of aglycone moieties, and rhamnose moieties, respectively. Geranic acid hexosyl-hexoside was mainly identified according to the characteristic fragments of a hexose moiety and [M + COOH]^−^ (*m/z* 537.2195), together with the fragments of [M – H]^−^ (*m/z* 491.2125), and [Agl + Hex – H]^−^ (*m/z* 329.1599) (Table 1). It is worth mentioning that in two published literatures, four compounds with molecular formula C_21_H_34_O_11_ (*m/z* 462.2101) were tentatively identified as monoterpenol malonylated glucosides by UHPLC-Q-TOF-MS, but the characteristic fragmentations were not found (Hjelmeland et al., 2015; Godshaw et al., 2019). We suggested that those compounds could be geranic acid pentosyl-hexosides, and the fragment at *m/z* 167.1075 indicated the loss of the sugar rings [also labeled as [Agl–H]^−^] in this study. This suggestion was in agreement with the viewpoint of other researchers (Flamini et al., 2018).

No monoterpenol hexoside was identified in the present assay. Certainly, the existence of mono-glycosides of monoterpenols in grape berry is still controversial (Hjelmeland et al., 2015; Godshaw et al., 2019). Caffrey et al. proposed that the previously reported monoterpenol hexoside should be the fragments of monoterpenol pentosyl-hexoside produced under very high fragmentor voltage (230 V) (Caffrey et al., 2020). In our experiment, the fragmentor voltage was set at a middle value (175 V), and no similar artifact of fragmentation appeared (Table 1). Monoterpene-polyols like monoterpene-triols and tetraols were not identified in this study although they have been reported in grape berries recently (Caffrey et al., 2020; Cebrian-Tarancon et al., 2021). One possible dihydromonoterpenetriol pentosyl-hexoside was also reported last year (Cebrian-Tarancon et al., 2021). Although no aglycone fragment was found, no discernible fragment can unequivocally identify this glycosidic compound. In general, only a few relevant studies on monoterpene-polyols and dihydromonoterpene-polyols have been reported, and the contribution of monoterpene-polyols to grape and wine aroma has not been widely studied.

#### Norisoprenoid Glycosides

**Figure 2:**
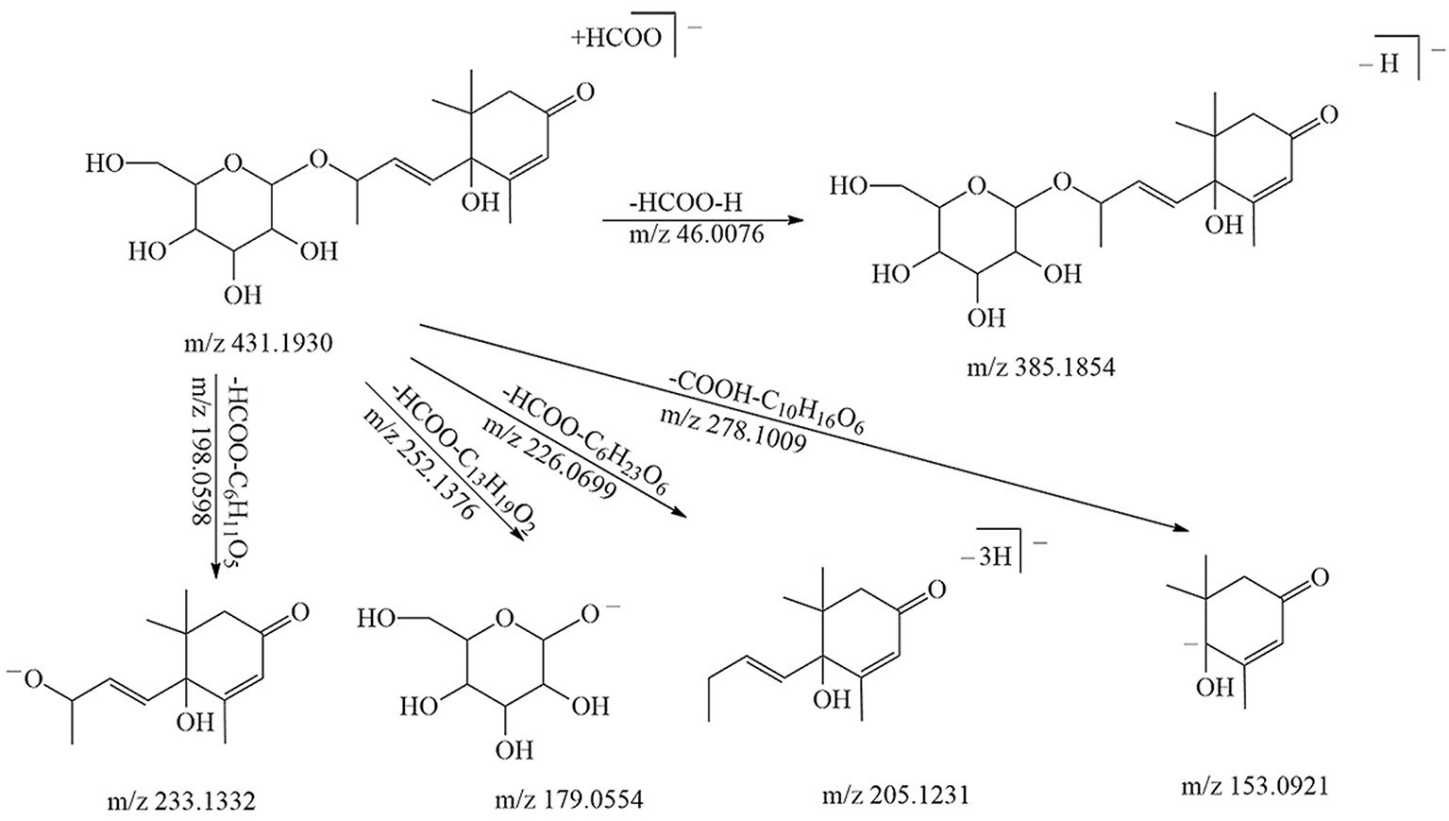
Fragmentation pattern of a vomifoliol hexoside.

Both norisoprenoid mono- and diglycosides were tentatively identified. The precursor ion of vomifoliol hexoside was [M + COOH]^−^ (*m/z* 431.1930), which was further fragmented into [M – H]^−^ (*m/z* 385.1854), [Hex – H]^−^ (*m/z* 179.0554), and [Agl – H]^−^ (*m/z* 205.1231) (Figure 2). The precursor ion of vomifoliol pentosyl-hexoside was found to be [M – H]^−^ at *m/z* 517.2288, and the compound was further deduced from the fragment ion [Agl – H]^−^ (*m/z* 205.1231), [Pen – H]^−^, [Hex – H]^−^, and [Pen + Hex – H – H_2_O]^−^. Similarly, the characteristic fragment ions of vomifoliol rhamnosyl-hexoside were presented as [M + COOH]^−^ at *m/z* 577.2499, together with the fragments of sugar ring moieties (*m/z* 307.1044 and *m/z* 325.1126). 3-Oxo-α-ionol and 3-hydroxy-β-damascenone were the isomers with the same molecular formula C_13_H_20_O_2_ (MW = 208.2970) and difficult to be distinguished just based upon the MS/MS information. Here, we temporarily named it as 3-oxo-α-ionol/3-hydroxy-β-damascenone. The pentosyl-hexoside of this aglycone was tentatively identified based on the fragment ions at *m/z* 547.2396 and *m/z* 311.0984 (Table 1). The rhamnosyl-hexoside of this aglycone displayed a similar fragmentation pattern with vomifoliol rhamnosyl-hexoside (Table 1).

Norisoprenoids are the important aroma contributors, especially in grape berries and wines of neutral variety as their concentration usually exceeds the sensory threshold (Mendes-Pinto, 2009). At present, only a few studies assessed norisoprenoid glycosides directly by LC-MS (Ghaste et al., 2015; Caffrey et al., 2020). Both vomifolyl glucoside and pentosyl-glucoside were assessed by LC-MS, and the first one had a high correlation with vomifoliol aglycone quantified by GC-MS after enzymatic hydrolysis (Ghaste et al., 2015). Other aglycones of norisoprenoid glycosides have been also found in grape leaves (Skouroumounis and Winterhalter, 1994) and berries (Caffrey et al., 2020), but the similar reports are still very limited. These compounds were not found in this research either.

#### Benzenoid Glycosides

The present approach identified seven benzenoid glycosides (Table 1). Benzyl alcohol pentosyl-hexoside had a similar structure with β-phenylethanol pentosyl-hexoside. The deprotonated benzyl alcohol pentosyl-hexoside [M – H]^−^ (*m/z* 401.1457) could be fragmented into [Agl + Hex – H]^−^ (*m/z* 269.1024) and a pentosyl-hexosyl moiety. β-Phenylethanol pentosyl-hexoside was tentatively deduced from the precursor ion [M – H]^−^ at *m/z* 415.1600. Meanwhile, this deduction was from [Agl + Hex – H]^−^ at *m/z* 283.1168 and the fragment ions of hexose and pentose moieties (Table 1). The precursor ion of β-phenylethanol rhamnosyl-hexoside was [M + COOH]^−^ (*m/z* 475.1823) and was further broken into [M – H]^−^ at *m/z* 429.1767 and [Rhm – H]^−^ (*m/z* 163.0616) (Supplementary Table 2). Interestingly, benzyl alcohol hexoside presented a special adduct ion form [2M – H]^−^, which was different from other compounds described above. Ions at *m/z* 177.0553 and *m/z* 195.0659 were assumed to be the fragments from aglycone moiety, and the hexose moiety was also observed (Supplementary Table 2). Previous study indicated that both benzyl alcohol and β-phenylethanol concentrations after enzymatic hydrolysis was highly correlated with the sum of their glycosidic forms measured by UHPLC-Q-TOF-high definition mass spectrometry (HDMS) (Ghaste et al., 2015). Benzyl alcohol and β-phenylethanol are the two common volatile phenols, and their glycosyl forms have been found to be monosaccharide, disaccharide, and trisaccharide glycosides in grape berries (Williams et al., 1983; Caffrey et al., 2020). In this research, we only identified monosaccharide. In contrast, Caffrey et al. observed volatile phenol trisaccharide but no monosaccharide glycoside, and they explained that the monosaccharide forms were possibly present but did not ionize efficiently in their study (Caffrey et al., 2020). Based on the ion [M + COOH]^−^ (*m/z* 505.1568) and its fragment ions [M – H]^−^ (*m/z* 459.1501), [Pen + Hex – H]^−^ (*m/z* 307.1049), and [Agl – H]^−^ (*m/z* 151.0402), it was postulated to be methyl salicylate pentosyl-hexoside, which was identified from the enzymatic hydrolysis product in grapes before (Fernandez-Gonzalez and Di Stefano, 2004). For the first time, the intact structure for methyl salicylate glycoside was tentatively identified in grapes.

#### C6/C9 Compound Glycosides

C6/C9 alcohols are biosynthesized from an oxylipin pathway and can be metabolized into C6/C9 aldehydes and esters (Lin et al., 2019). These C6/C9 compounds are classified as “Green Leaf Volatiles” (GLVs) with “green” and “fresh” odor. The concentration of 1-hexanol and 3-hexen-1-ol as well as the ratios between them can be used as an indicator of the variety of origin, so that they are supposed to be the most important C6 compounds in grapes and wine (Oliveira et al., 2006). More importantly, the content of some C6 compounds especially hexyl acetate in wine was demonstrated to depend on their glycosidic precursor in berries (Keyzers and Boss, 2010). Six C6/C9 compound glycosides were identified in this study. 3-Hexen-1-ol hexosyl-hexoside was confirmed according to its precursor ion [M – H]^−^ (*m/z* 423.1866) that was further fragmented into a 3-hexen-1-ol hexosyl moiety (*m/z* 261.1340) (Table 1), which was in line with two recent studies (Caffrey et al., 2020; Cebrian-Tarancon et al., 2021). A 3-hexen-1-ol trisaccharide was also tentatively identified, and a similar compound C6-alkoxy-trisaccharide has been found in grapes (Caffrey et al., 2020). The fragment signal at *m/z* 441.1978 and *m/z* 409.2069 were inferred to be [M + COOH]^−^ and [M – H]^−^ of 1-hexanol pentosyl-hexoside, and the fragments of [Agl + Hex – H]^−^ (*m/z* 263.1496) and sugar moieties were also observed (Table 1, Supplementary Table 2). 1-Hexanol rhamnosyl-hexoside was confirmed by the similar fragmentation pattern. 1-Nonanol pentosyl-hexoside was identified based on [M + COOH – H_2_O]^−^ (*m/z* 465.2332) and the typical fragment ions of pentosyl-hexosyl moiety (*m/z* 293.0870).

#### Other Compounds

Furaneol is generally described as having a strawberry, pineapple or raspberry note with low-sensory threshold. In this study, only furaneol pentosyl-hexoside was tentatively identified with a precursor ion of [M – H]^−^. A unique deprotonated signal of furaneol rhamnosyl-glucoside was previously found, and its peak area was highly correlated with the furaneol content by GC-MS (Ghaste et al., 2015). Another compound was confirmed as 3-methyl-1-butanol pentosyl-hexoside based on its precursor ions [M – H]^−^ (*m/z* 381.1771) together with *m/z* 249.1345. Five alcohol glycosides conforming to the fragmentation patterns were screened out by ChemSpider online database. Isopropyl alcohol pentosyl-hexoside was tentatively identified in a form of [M – H]^−^, which could be further fragmented into [Agl + Hex – H]^−^and [Hex – H – H_2_O]^−^. Pentosyl-hexosides of 3-methyl-2-buten-1-ol, 2-butanol, and 1,10-decanediol showed similar fragmentation patterns, and the fragment ions of [Agl + Hex – H]^−^ and sugar ring moieties are shown in Table 1.

### Difference of Glycosidic Aroma Compounds Among Three Grape Varieties

**Figure 3:**
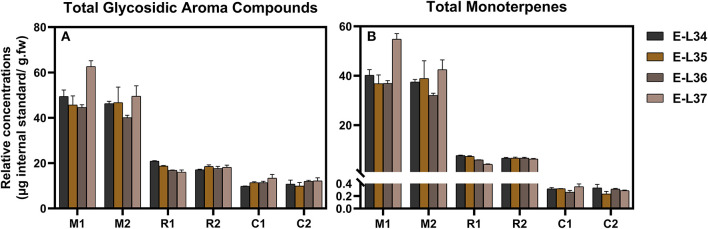
Relative concentrations of total glycosidic compounds **(A)** and total monoterpenes **(B)** in three grape varieties (two clones of each) at four ripening stages. M1, M2, R1, R2, C1, and C2 correspond to the clone 1 and clone 2 of Muscat Blanc (M), Riesling (R), and Chardonnay (C), respectively, as described in detail in the Plant materials section. Different colors are used for four ripening stages. The original data used were shown in Supplementary Table 3.

**Figure 4:**
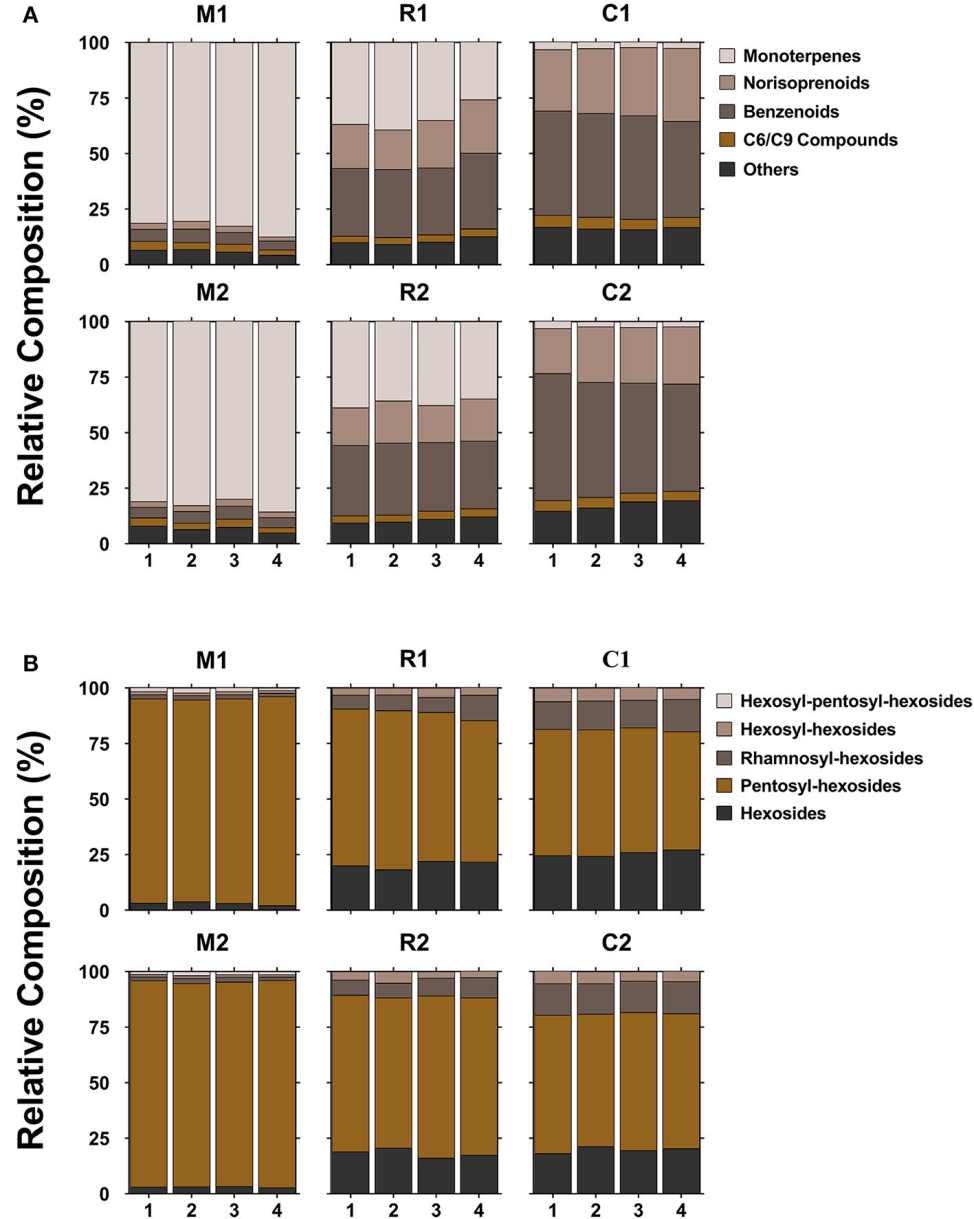
The proportion of each aglycone class **(A)** and glycone type **(B)** of glycosidic compounds to the total in three grape varieties (two clones of each) at four ripening stages. 1, 2, 3, and 4 refer to E-L34, E-L35, E-L36, and E-L37, respectively. The marks below refer to the statement shown in Figure 3. Different colors are used for five aglycone or glycone classes.

Samples from the three varieties (two clones per variety) were chosen for this method application. A total of 46 compounds were tentatively identified, but only 17 compounds were shared by all the three varieties. The mean values of concentration of various compounds with respect to the internal standard were shown in Supplementary Table 3. Due to the lack of standards, it is difficult to analyze the absolute abundances of each glycoside found in Supplementary Table 3, but relative abundances were suitable for the comparison of the same compounds among varieties and ripening stages. It was observed that two clones of the same variety possessed similar compositions and concentrations of glycosidic compounds; whereas, the three varieties displayed a large difference. This profiling pattern may be associated with the grape varietal characteristics itself. According to a general classification of winemaking grapes (Mateo and Jimenez, 2000), Muscat Blanc is considered to be a Muscat type variety with a high level of free-form monoterpenes, Riesling belongs to a non-Muscat aromatic variety, and Chardonnay is a neutral variety. Muscat Blanc had the most abundant glycosidic aroma compounds in either types or concentration, followed by Riesling and Chardonnay (Figure 3A, Supplementary Table 3). This meant that Muscat-type variety contained high levels of not only free-form monoterpenes but also glycosidic aroma compounds. The sum of monoterpene glycosides in Chardonnay was much lower than those in Muscat Blanc or Riesling (Figure 3B, Supplementary Table 3) and in line with previous study (Godshaw et al., 2019). Neutral varieties generally contained a very low concentration of monoterpene glycosides (Nasi et al., 2008). Based on the aglycone classes, the proportion of each class to the total is shown in Figure 4A. In terms of concentration proportion, monoterpene glycosides were the main glycosidic aroma compounds in ripe Muscat Blanc berries (Figure 4A). In a similar study on Muscat of Alexandria, the two-third of the glycosidic aroma compounds identified was monoterpenes (Cebrian-Tarancon et al., 2021). The norisoprenoid and benzenoid glycosides accounted for a substantial part of glycosidic aroma compounds in Chardonnay, and the glycosides of monoterpenes, norisoprenoids and benzenoids, respectively, had similar proportions in Reisling. Most neutral varieties are not dependent on free-form monoterpenes for their flavor (Mateo and Jimenez, 2000). It seems that monoterpene glycosides barely contribute to aroma profile of neutral varieties. By contrast, norisoprenoids are regarded as the varietal aroma compounds of neutral grape varieties such as Chardonnay, Cabernet Sauvignon, and others (Meng et al., 2020). The present study indicated that norisoprenoid glycosides, next to benzenoid glycosides, took up a certain concentration proportion in Chardonnay berries (Figure 4A). Glycosidic aroma compounds were divided into five classes according to the types of glycones (Figure 4B). Diglycosides especially pentosyl-hexosides were the most abundant in all the three varieties. Almost every type of aglycones, with the exception of 3-oxo-α-ionol/3-hydroxy-β-damascenone, had pentosyl-hexosidic form in grapes (Table 1). In the three varieties, trisaccharide glycosides were found to only band with monoterpene-diols and monoterpenols (Table 1). Moreover, only Muscat Blanc had a small amount of trisaccharide glycosides (compounds 9–11 and 20).

**Figure 5:**
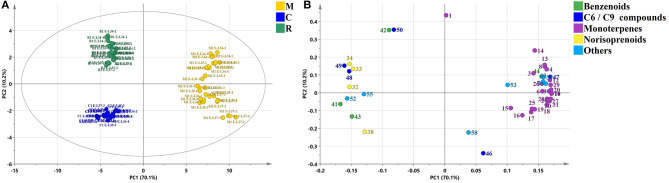
Principal component analysis **(A)** and component loading plot **(B)** of glycosidic aroma compounds from three grape varieties (two clones of each). The marks in plot **(A)** refer to the statement shown in Figure 3. The numbers in plot **(B)** correspond to compounds mentioned in Table 1, Supplementary Tables 2, 3, Figures 1, 6.

It was known that major volatile compounds of berries from fruit set to harvest can significantly discriminate varieties (Kalua and Boss, 2010). To further differentiate the profiling of glycosidic aroma compounds across the three varieties, PCA of 46 compounds were applied in Figure 5 (*R*^2^*X* = 0.803, *Q*^2^ = 0.764). The two principal components explained 80.3% of the variance among the three different varieties. The first principal component (PC1) explained 70.1% of the total variance and the second principal component (PC2) of 10.2%. Both clones of Muscat Blanc were localized in the positive direction of PC1, while Riesling and Chardonnay were in the negative direction of PC1 (Figure 5A), and they could be clearly separated from each other by PC2 (Figure 5A). From the component loading plot (Figure 5B), it was seen that almost all monoterpene glycosides, with the exception of compounds 1, were concentrated in the positive PC1, indicating that Muscat Blanc was characterized by abundant monoterpene glycosides. Similarly, glycosides of norisoprenoids and benzenoids mainly contributed to the Riesling and Chardonnay as most of these compounds appeared in the negative PC1. Clearly, Muscat-type variety could be differentiated from non-Muscat varieties with the profiling of monoterpene glycosides, so to non-Muscat varieties with glycosides of norisoprenoids and benzenoids. Compounds 1, 42, and 50 were clustered in the positive direction of PC2, which were related to Riesling. Compound 1, being a monoterpene-diol pentosyl-hexoside, displayed a higher concentration in Riesling compared to Muscat Blanc; whereas, all other monoterpene-diol pentosyl-hexosides detected in this assay had the highest concentrations in Muscat Blanc (Supplementary Table 3). In a recent study, one compound, which was marked as monoterpene-diol pentosyl-hexoside 1, also had the highest concentration in Riesling compared to other detected grape varieties (Godshaw et al., 2019). Compounds 46 and 58 were associated with Chardonnay in the negative direction of PC2 (Figure 5B). It seems that C6/C9 compounds could be used to distinguish non-Muscat aromatic varieties and neutral varieties.

### Change of Glycosidic Aroma Compounds During Grape Maturation

**Figure 6:**
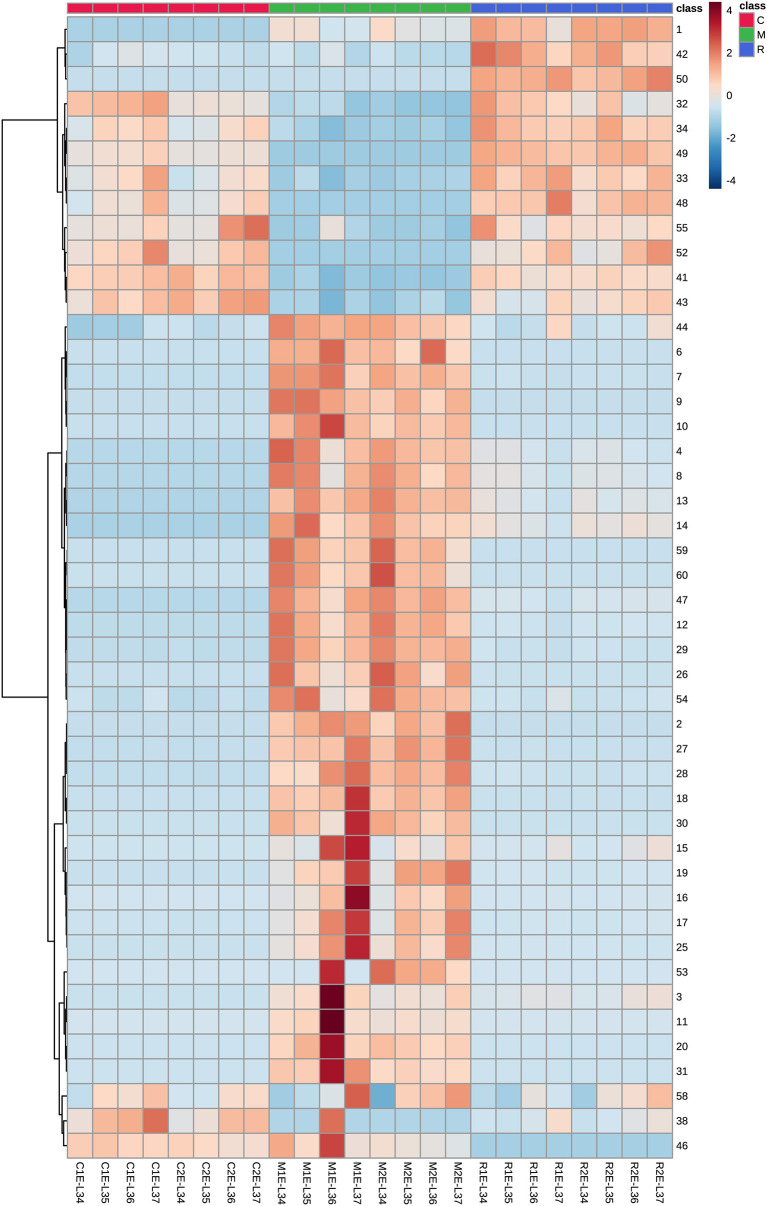
Heatmap of the transformed data for various glycosidic aroma compounds in three grape varieties at four ripening stages. The marks below refer to the statement shown in Figure 3. The numbers on the right correspond to compounds mentioned in Table 1, Supplementary Tables 2, 3, Figures 1, 5.

Glycosidic aroma compounds in the three varieties at four stages before (E-L 34) and after veraison (E-L 36, E-L 37) were analyzed. The relative ripening index was shown in Supplementary Table 4. It was shown that the composition of glycosides kept unchanged, and the concentrations of most glycosides kept relatively stable during the veraison (Figure 3, Supplementary Table 3). In Moscato bianco, only 5 out of 12 glycosidic terpenes have shown significant differences during ripening (Torchio et al., 2016). Heatmap was used to visualize the accumulation patterns of glycosidic aroma compound during berry maturation (Figure 6). The concentration of some monoterpene disaccharide glycosides (compounds 2 and 15–19), geranic acid, and citronellol glycosides (compounds 25, 27, 28, and 30) remarkably increased after veraison in both clones of Muscat Blanc but kept unchanged in another two varieties. In contrast, the other monoterpene disaccharide glycosides (compounds 4, 8, 12–14, 26, and 29) decreased after veraison in Muscat Blanc berries and almost unchanged in Riesling and Chardonnay. The meta-analysis of the aroma compounds of grape and wine aroma showed that concentrations of some monoterpenes are tightly correlated, which indicates they have common metabolic origin (Ilc et al., 2016). However, it was still difficult to find a certain pattern based on aglycone or glycone structures to explain. In two different clones of Muscat Blanc, several compounds showed different variations with berry maturation. Compounds 3, 11, 20, and 31 exhibited an increasing trend after veraison in the berries from M1while those compounds did not show remarkable change in M2 (Figure 6). Given M1 and M2 had the same rootstock 5BB, this discrepant accumulation pattern may be attributed to different scions. Norisoprenoid glycosides (compounds 32–38) were accumulated with berry maturation, and their increases were more pronounced in C1 than C2 (Figure 6). A previous study revealed that norisoprenioid glycosides elevated during veraison and 3-oxo-α-ionol were the major norisoprenioid glycoside components in Shiraz and Muscat of Alexandria berries (Mathieu et al., 2005). Benzyl alcohol and β-phenylethanol were reported as the most abundant glycosidic aroma compounds in ripe berries (Vilanova et al., 2012). Due to the lack of standards, it is difficult to compare the absolute concentrations among compounds; however, it is possible to analyze the accumulation pattern of glycosidic benzyl alcohol and β-phenylethanol during ripening. The concentrations of these two important benzenoids were reported to be relatively stable during ripening (Fenoll et al., 2009). A similar situation was observed in our study (Figure 6).

## Conclusions

In this study, we constructed a mass spectral database consisting of 60 glycosidic aroma compounds through the semi-qualitative analysis of 10 grape varieties for the UHPLC-Q-TOF-MS analysis. The database covered multiple aglycone classes including monoterpene, norisoprenoid, benzenoids, C6/C9 compound, and other alcohols. Profiling of glycosidic aroma compounds was investigated in six clones of three grape varieties at four maturation stages. The most abundant glycosylation pattern in grapes is pentosyl-hexoside. This study indicates that not only monoterpenes but also other aglycones exist in the form of triglycosides. The glycosylation patterns of aroma compounds display a remarkable difference among varieties, and the difference is mainly in the compound concentration between clones of same variety. Muscat Blanc variety is characterized by abundant types and high concentrations of monoterpene glycosides, and Riesling and Chardonnay by norisoprenoid and benzenoid glycosides. Except for monoterpenol pentosyl-hexosides, most of the glycosidic aroma components did not show a noticeable concentration variation during berry maturation. In summary, this study provided an approach to understand the glycosidic aroma compounds especially glycones and their accumulating patterns in grape berries under ripening stages. Combining with molecular biological investigation, one can explore the regulation of glycosidic aroma compound biosynthesis in grape berries.

## Data Availability Statement

The original contributions presented in the study are included in the article/Supplementary Material, further inquiries can be directed to the corresponding author/s.

## Author Contributions

YW interpreted the data and wrote the manuscript. ZC and X-KZ performed the experiments and assisted in the interpretation of the data. C-QD and Q-HP designed the experiments. All authors contributed to the article and approved the submitted version.

## Conflict of Interest

The authors declare that the research was conducted in the absence of any commercial or financial relationships that could be construed as a potential conflict of interest.

## References

[B1] Caffrey A. Lerno L. Rumbaugh A. Girardello R. Zweigenbaum J. Oberholster A.. (2019). Changes in smoke-taint volatile-phenol glycosides in wildfire smoke-exposed Cabernet Sauvignon grapes throughout winemaking. Am. J. Enol. Viticult. 70, 373–381. 10.5344/ajev.2019.19001

[B2] Caffrey A. J. Lerno L. A. Zweigenbaum J. Ebeler S. E. (2020). Direct analysis of glycosidic aroma precursors containing multiple aglycone classes in *Vitis vinifera* berries. J. Agr. Food Chem. 68, 3817–3833. 10.1021/acs.jafc.9b08323 32129620

[B3] Cebrian-Tarancon C. Oliva J. Camara M. A. Alonso G. L. Salinas M. R. (2021). Analysis of intact glycosidic aroma precursors in grapes by high-performance liquid chromatography with a diode array detector. Foods 10:191. 10.3390/foods10010191 33477839 PMC7832828

[B4] Chong J. Wishart D. S. Xia J. (2019). Using metaboanalyst 4.0 for comprehensive and integrative metabolomics data analysis. Curr. Protoc. Bioinformatics. 68:e86. 10.1002/cpbi.86 31756036

[B5] Coombe B. G. (1995). Adoption of a system for identifying grapevine growth stages. Aust. J. Grape Wine R. 1, 104–110. 10.1111/j.1755-0238.1995.tb00086.x

[B6] Fenoll J. Manso A. Hellin P. Ruiz L. Flores P. (2009). Changes in the aromatic composition of the *Vitis vinifera* grape Muscat Hamburg during ripening. Food Chem. 114, 420–428. 10.1016/j.foodchem.2008.09.060

[B7] Fernandez-Gonzalez M. Di Stefano R. (2004). Fractionation of glycoside aroma precursors in neutral grapes. Hydrolysis and conversion by Saccharomyces cerevisiae. Lebensm Wiss Technol. 37, 467–473. 10.1016/j.lwt.2003.11.003

[B8] Flamini R. De Rosso M. Panighel A. Dalla Vedova A. De Marchi F. Bavaresco L. (2014). Profiling of grape monoterpene glycosides (aroma precursors) by ultra-high performance-liquid chromatography-high resolution mass spectrometry (UHPLC/QTOF). J. Mass Spectrom. 49, 1214–1222. 10.1002/jms.3441 25476938

[B9] Flamini R. Menicatti M. De Rosso M. Gardiman M. Mayr C. Pallecchi M.. (2018). Combining liquid chromatography and tandem mass spectrometry approaches to the study of monoterpene glycosides (aroma precursors) in wine grape. J. Mass Spectrom. 53, 792–800. 10.1002/jms.4212 29907998

[B10] Gao Y. Li X. X. Han M. M. Yang X. F. Li Z. Wang J.. (2016). Rain-shelter cultivation modifies carbon allocation in the polyphenolic and volatile metabolism of *Vitis vinifera* L. Chardonnay grapes. PLoS ONE. 11:e0156117. 10.1371/journal.pone.0156117 27218245 PMC4878772

[B11] Ghaste M. Narduzzi L. Carlin S. Vrhovsek U. Shulaev V. Mattivi F. (2015). Chemical composition of volatile aroma metabolites and their glycosylated precursors that can uniquely differentiate individual grape cultivars. Food Chem. 188, 309–319. 10.1016/j.foodchem.2015.04.056 26041197

[B12] Godshaw J. Hjelmeland A. K. Zweigenbaum J. Ebeler S. E. (2019). Changes in glycosylation patterns of monoterpenes during grape berry maturation in six cultivars of *Vitis vinifera*. Food Chem. 297, 124921–124921. 10.1016/j.foodchem.2019.05.195 31253264

[B13] Gunata Y. Z. Bayonove C. L. Baumes R. L. Cordonnier R. E. (1985a). The aroma of grapes—localization and evolution of free and bound fractions of some grape aroma components cv Muscat during 1st development and maturation. J. Sci. Food Agr. 36, 857–862. 10.1002/jsfa.2740360915

[B14] Gunata Y. Z. Bayonove C. L. Baumes R. L. Cordonnier R. E. (1985b). The aroma of grapes I. Extraction and determination of free and glycosidically bound fractions of some grape aroma components. J. Chromatogr. A 331, 83–90. 10.1016/0021-9673(85)80009-1

[B15] Guth H. (1997). Quantitation and sensory studies of character impact odorants of different white wine varieties. J. Agr. Food Chem. 45, 3027–3032. 10.1021/jf970280a

[B16] Hjelmeland A. K. Ebeler S. E. (2015). Glycosidically bound volatile aroma compounds in grapes and wine: a review. Am. J. Enol. Viticult. 66, 1–11. 10.5344/ajev.2014.14104

[B17] Hjelmeland A. K. Zweigenbaum J. Ebeler S. E. (2015). Profiling monoterpenol glycoconjugation in *Vitis vinifera* L. cv. Muscat of Alexandria using a novel putative compound database approach, high resolution mass spectrometry and collision induced dissociation fragmentation analysis. Anal. Chim. Acta. 887, 138–147. 10.1016/j.aca.2015.06.026 26320795

[B18] Ilc T. Werck-Reichhart D. Navrot N. (2016). Meta-analysis of the core aroma components of grape and wine aroma. Front. Plant Sci. 7:1472. 10.3389/fpls.2016.01472 27746799 PMC5042961

[B19] Kalua C. M. Boss P. K. (2010). Comparison of major volatile compounds from Riesling and Cabernet Sauvignon grapes (*Vitis vinifera* L.) from fruitset to harvest. Aust. J. Grape Wine R. 16, 337–348. 10.1111/j.1755-0238.2010.00096.x

[B20] Keyzers R. A. Boss P. K. (2010). Changes in the volatile compound production of fermentations made from musts with increasing grape content. J. Agr. Food Chem. 58, 1153–1164. 10.1021/jf9023646 20020683

[B21] Lan Y. B. Xiang X. F. Qian X. Wang J. M. Ling M. Q. Zhu B. Q.. (2019). Characterization and differentiation of key odor-active compounds of 'Beibinghong' icewine and dry wine by gas chromatography-olfactometry and aroma reconstitution. Food Chem. 287, 186–196. 10.1016/j.foodchem.2019.02.074 30857688

[B22] Lin J. Massonnet M. Cantu D. (2019). The genetic basis of grape and wine aroma. Hortic. Res. England. 6:81. 10.1038/s41438-019-0163-1 31645942 PMC6804543

[B23] Loscos N. Hernandez-Orte P. Cacho J. Ferreira V. (2009). Comparison of the suitability of different hydrolytic strategies to predict aroma potential of different grape varieties. J. Agr. Food Chem. 57, 2468–2480. 10.1021/jf803256e 19231895

[B24] Mateo J. J. Jimenez M. (2000). Monoterpenes in grape juice and wines. J. Chromatogr. A 881, 557–567. 10.1016/S0021-9673(99)01342-4 10905735

[B25] Mathieu S. Terrier N. Procureur J. Bigey F. Gunata Z. (2005). A carotenoid cleavage dioxygenase from *Vitis vinifera* L.: functional characterization and expression during grape berry development in relation to C-13-norisoprenoid accumulation. J. Exp. Bot. 56, 2721–2731. 10.1093/jxb/eri265 16131507

[B26] Mendes-Pinto M. M. (2009). Carotenoid breakdown products the—norisoprenoids—in wine aroma. Arch. Biochem. Biophys. 483, 236–245. 10.1016/j.abb.2009.01.008 19320050

[B27] Meng N. Wei Y. Gao Y. Yu K. Cheng J. Li X. Y.. (2020). Characterization of transcriptional expression and regulation of carotenoid cleavage dioxygenase 4b in grapes. Front. Plant Sci. 11:483. 10.3389/fpls.2020.00483 32457771 PMC7227400

[B28] Metafa M. Economou A. (2013). Comparison of solid-phase extraction sorbents for the fractionation and determination of important free and glycosidically-bound varietal aroma compounds in wines by gas chromatography-mass spectrometry. Cent. Eur. J. Chem. 11, 228–247. 10.2478/s11532-012-0154-7

[B29] Nasi A. Ferranti P. Amato S. Chianese L. (2008). Identification of free and bound volatile compounds as typicalness and authenticity markers of non-aromatic grapes and wines through a combined use of mass spectrometric techniques. Food Chem. 110, 762–768. 10.1016/j.foodchem.2008.03.001

[B30] Oliveira J. M. Faria M. Sa F. Barros F. Araujo I. A. (2006). C-6-alcohols as varietal markers for assessment of wine origin. Anal. Chim. Acta. 563, 300–309. 10.1016/j.aca.2005.12.029

[B31] Parker M. Capone D. L. Francis I. L. Herderich M. J. (2018). Aroma precursors in grapes and wine: flavor release during wine production and consumption. J. Agr. Food Chem. 66, 2281–2286. 10.1021/acs.jafc.6b05255 28220693

[B32] Sarry J. E. Gunata Z. (2004). Plant and microbial glycoside hydrolases: volatile release from glycosidic aroma precursors. Food Chem. 87, 509–521. 10.1016/j.foodchem.2004.01.003

[B33] Schievano E. D'Ambrosio M. Mazzaretto I. Ferrarini R. Magno F. Mammi S.. (2013). Identification of wine aroma precursors in Moscato Giallo grape juice: a nuclear magnetic resonance and liquid chromatography-mass spectrometry tandem study. Talanta 116, 841–851. 10.1016/j.talanta.2013.07.049 24148483

[B34] Skouroumounis G. K. Sefton M. A. (2000). Acid-catalyzed hydrolysis of alcohols and their beta-d-glucopyranosides. J. Agr. Food Chem. 48, 2033–2039. 10.1021/jf9904970 10888494

[B35] Skouroumounis G. K. Winterhalter P. (1994). Glycosidically bound norisoprenoids from *Vitis-vinifera* cv Riesling leaves. J. Agr. Food Chem. 42, 1068–1072. 10.1021/jf00041a004

[B36] Torchio F. Giacosa S. Vilanova M. Segade S. R. Gerbi V. Giordano M.. (2016). Use of response surface methodology for the assessment of changes in the volatile composition of Moscato Bianco (*Vitis vinifera* L.) grape berries during ripening. Food Chem. 212, 576–584. 10.1016/j.foodchem.2016.05.191 27374570

[B37] Verardo G. Duse I. Callea A. (2009). Analysis of underivatized oligosaccharides by liquid chromatography/electrospray ionization tandem mass spectrometry with post-column addition of formic acid. Rapid Commun. Mass Spectrom. 23, 1607–1618. 10.1002/rcm.4047 19408275

[B38] Vilanova M. Genisheva Z. Bescansa L. Masa A. Oliveira J. M. (2012). Changes in free and bound fractions of aroma compounds of four *Vitis vinifera* cultivars at the last ripening stages. Phytochemistry 74, 196–205. 10.1016/j.phytochem.2011.10.004 22071134

[B39] Voirin S. Baumes R. Bayonove C. Mbairaroua O. Tapiero C. (1990). Synthesis and n.m.r. spectral properties of grape monoterpenyl glycosides. Carbohyd. Res. 207, 39–56. 10.1016/0008-6215(90)80004-M

[B40] Voirin S. G. Baumes R. L. Sapis J. C. Bayonove C. L. (1992). Analytical methods for monoterpene glycosides in glycosides in grape and wine. II. Qualitative and quantitative-determination of monoterpene glycosides in grape. J. Chromatogr. 595, 269–281. 10.1016/0021-9673(92)85169-T 1577909

[B41] Wang Y. Li H. Q. Gao X. T. Lu H. C. Peng W. T. Chen W.. (2020). Influence of attenuated reflected solar radiation from the vineyard floor on volatile compounds in Cabernet Sauvignon grapes and wines of the north foot of Mt. Tianshan. Food Res. Int. 137:109688. 10.1016/j.foodres.2020.109688 33233263

[B42] Williams P. J. Strauss C. R. Wilson B. Massywestropp R. A. (1983). Glycosides of 2-phenylethanol and benzyl alcohol in *Vitis-vinifera* grapes. Phytochemistry 22, 2039–2041. 10.1016/0031-9422(83)80040-5

[B43] Wilson B. Strauss C. R. Williams P. J. (1984). Changes in free and glycosidically bound monoterpenes in developing Muscat grapes. J. Agr. Food Chem. 32, 919–924. 10.1021/jf00124a054

[B44] Winterhalter P. Skouroumounis G. K. (1997). Glycoconjugated aroma compounds: occurrence, role and biotechnological transformation. Adv. Biochem. Eng. Biotechnol. 55, 73–105. 10.1007/BFb0102063 9017925

[B45] Yang Y. Jin G. J. Wang X. J. Kong C. L. Liu J. Tao Y. S. (2019). Chemical profiles and aroma contribution of terpene compounds in Meili (*Vitis vinifera* L.) grape and wine. Food Chem. 284, 155–161. 10.1016/j.foodchem.2019.01.106 30744840

