# Supplementary material for: Comparative Analysis of Glycosidic Aroma Compound Profiling in Three Vitis vinifera Varieties by Using Ultra-High-Performance Liquid Chromatography Quadrupole-Time-of-Flight Mass Spectrometry
Source: Front Plant Sci. 2021 Jun 24;12:694979. doi: 10.3389/fpls.2021.694979 (PMC8264444; doi:10.3389/fpls.2021.694979)
Supplement: Supplementary file 1 [file Table_1.docx]

**Supplementary Table 1** Identification of glycosidic volatile components in 10 grape varieties using GC-MS. +: detected, −: not detected.

| **Compounds** | **CAS** | **M1** | **M2** | **C1** | **C2** | **R1** | **R2** | **GWU** | **MAN** | **ROU** | **ARA** | **VIO** | **ITAR** | **SAUB** |
| --- | --- | --- | --- | --- | --- | --- | --- | --- | --- | --- | --- | --- | --- | --- |
| **Benzenoids** |  |  |  |  |  |  |  |  |  |  |  |  |  |  |
| Benzaldehyde | 100-52-7 | − | − | − | + | + | + | − | − | − | − | − | − | − |
| 2,4-Dimethyl-benzaldehyde | 15764-16-6 | + | + |  | + | + | + | + | + | + | + | − | − | + |
| 2,5-Dimethyl-benzaldehyde | 5779-94-2 | − | − | − | − | − | − | − | − | − | − | + | − | − |
| 4-Methyl-benzaldehyde | 104-87-0 | − | − | − | − | − | − | − | − | − | − | − | + | − |
| 1-Ethyl-4-methyl-benzene | 622-96-8 | − | − | − | + | + | − | − | − | − | − | − | − | − |
| 1-Methyl-4-(1-methylethenyl)-benzene | 1195-32-0 | − | − | − | − | − | − | + | − | − | − | − | + | − |
| Benzyl alcohol | 100-51-6 | + | + | + | + | + | + | + | + | + | + | + | − | + |
| Methyl salicylate | 119-36-8 | − | − | − | − | − | − | − | − | − | − | + | − | − |
| Phenylethyl alcohol | 60-12-8 | + | + | + | + | + | + | + | + | + | + | + | − | + |
| *p*-Cymene | 99-87-6 | − | + | − | − | − | − | + | − | − | − | − | + | − |
| **Alcohols** |  |  |  |  |  |  |  |  |  |  |  |  |  |  |
| 3-Methyl-1-butanol | 123-51-3 | − | − | − | − | − | − | − | − | − | − | + | − | − |
| 1-Heptanol | 111-70-6 | − | − | − | − | − | − | − | − | − | − | + | − | + |
| 2-Ethyl-1-hexanol | 104-76-7 | + | + | + | + | + | + | + | + | + | + | + | + | + |
| 1-Octanol | 111-87-5 | + | + | + | + | + | + | + | + | + | + | + | + | − |
| 1-Octen-3-ol | 3391-86-4 | + | + | + | + | + | + | − | − | − | − | − | − | − |
| 2-Heptanol | 543-49-7 | + | + | + | + | + | + | − | − | + | − | + | − | − |
| 3-Octanol | 589-98-0 | + | − | + | − | + | − | − | − | − | − | − | − | − |
| **Ester** |  |  |  |  |  |  |  |  |  |  |  |  |  |  |
| Ethyl nonanoate | 123-29-5 | − | − | − | − | − | + | − | − | − | − | − | − | − |
| **Aldehydes** |  |  |  |  |  |  |  |  |  |  |  |  |  |  |
| Octanal | 124-13-0 | − | − | − | + | − | + | − | − | − | − | − | − | − |
| (*E*)-2-Octenal | 2548-87-0 | − | + | − | − | − | + | + | − | − | − | − | − | − |
| **Ketone** |  |  |  |  |  |  |  |  |  |  |  |  |  |  |
| 2-Hexanone | 591-78-6 | − | − | + | − | − | − | − | − | − | − | − | − | − |
| **Monoterpenes** |  |  |  |  |  |  |  |  |  |  |  |  |  |  |
| 2,6-Dimethyl-2,4,6-octatriene | 673-84-7 | + | + | − | − | − | − | − | − | − | − | − | − | − |
| (*E*,*Z*)-2,6-Dimethyl-2,4,6-octatriene | 7216-56-0 | + | + | − | − | − | − | + | − | − | − | − | − | − |
| 3,4-Dimethyl-2,4,6-octatriene | 57396-75-5 | − | − | − | − | − | − | + | − | − | − | − | − | − |
| *(Z)*-*β*-Ocimene | 3338-55-4 | + | + | − | − | − | − | + | − | − | − | − | − | − |
| *cis*-Linaloloxide | EPA-121974 | + | − | − | − | − | − | − | − | − | − | − | − | − |
| *cis*-Linalool-oxide | 5989-33-3 | − | + | − | − | + | + | − | − | − | − | − | − | − |
| Citral | 5392-40-5 | + | − | − | − | − | − | − | − | − | − | − | − | − |
| Geranial | 141-27-5 | + | + | − | − | − | − | + | − | − | − | − | − | − |
| Limonene | 138-86-3 | + | + | − | − | − | + | + | − | − | − | − | − | − |
| Neroloxide | 1786-08-9 | + | + | − | − | − | − | + | − | − | − | − | − | − |
| Rose oxide | 16409-43-1 | + | − | − | − | − | − | + | − | − | − | − | − | − |
| Terpinolene | 586-62-9 | + | + | − | − | − | − | − | − | − | − | − | − | − |
| *(E)*-*β*-Ocimene | 3779-61-1 | + | + | − | − | + | + | + | − | − | − | − | − | − |
| *trans*-Linalooloxide(furanoid) | 34995-77-2 | + | + | − | − | − | − | + | − | − | − | − | − | − |
| *α*-Terpinene | 99-86-5 | + | + | − | − | − | − | + | − | − | − | − | − | − |
| *α*-Terpineol | 98-55-5 | + | + | − | − | + | − | + | − | − | − | − | + | − |
| *β*-Linalool | 78-70-6 | + | + | − | − | + | − | + | − | − | − | − | − | − |
| *β*-Phellandrene | 555-10-2 | + | + | − | − | − | − | + | − | − | − | − | − | − |
| *γ*-Terpineol | 586-81-2 | − | + | − | − | − | − | − | − | − | − | − | − | − |
| **Norisoprenoids** |  |  |  |  |  |  |  |  |  |  |  |  |  |  |
| (*E*)-1-(2,3,6-trimethylphenyl)buta-1,3-diene(TPB) | EPA-357257 | − | − | − | − | − | − | − | − | − | − | − | + | + |
| *trans*-1-(2,6,6-trimethyl-1,3-cyclohexadien-1-yl)-2-buten-1-one(*trans*-*β*-damascenone) | 23696-85-7 | − | − | − | − | − | − | − | − | − | − | − | + | − |
| 6-Methyl-5-hepten-2-ol | 1569-60-4 | + | + | − | − | + | + | + | − | − | − | − | − | − |
| 6-Methyl-5-hepten-2-one | 110-93-0 | + | − | − | − | − | − | − | − | − | − | − | − | + |
| **C6/C9 compounds** |  |  |  |  |  |  |  |  |  |  |  |  |  |  |
| 1-Hexanol | 111-27-3 | + | + | + | + | + | + | + | + | + | + | + | + | + |
| 1-Nonanol | 143-08-8 | − | − | − | − | − | − | − | − | − | + | − | − | − |
| (*E*)-2-Hexen-1-ol | 928-95-0 | − | − | − | − | + | − | − | − | − | − | − | − | − |
| 2-Hexenal | 505-57-7 | − | − | − | − | − | + | − | − | − | − | − | − | − |
| (*E*)-2-Hexenal | 6728-26-3 | − | − | − | + | − | − | − | + | − | − | − | + | + |
| (*Z*)-3-Hexen-1-ol | 928-96-1 | − | − | + | − | − | − | − | − | − | + | − | − | − |
| Nonanal | 124-19-6 | + | + | + | + | + | + | + | + | + | + | + | + | + |

*M1, M2, R1, R2, C1, C2, GWU, MAN, ROU, ARA, VIO, TAR and AUB represent grape varieties, which are described in detail in “Materials and methods” section.*
