# Supplementary material for: Comparative Analysis of Glycosidic Aroma Compound Profiling in Three Vitis vinifera Varieties by Using Ultra-High-Performance Liquid Chromatography Quadrupole-Time-of-Flight Mass Spectrometry
Source: Front Plant Sci. 2021 Jun 24;12:694979. doi: 10.3389/fpls.2021.694979 (PMC8264444; doi:10.3389/fpls.2021.694979)
Supplement: Supplementary file 2 [file Table_2.docx]

**Supplementary Table 2** Fragmentation ion information of glycosidic aroma compounds identified in 10 grape varieties.

| **No.^*^** | **Compound** | **MS/MS Product Ions (*m/z*)** |
| --- | --- | --- |
| 1 | Monoterpene-diol pentosyl-hexoside-1 | 59.0144, 71.0143, 89.0243, 101.0245, 113.0242, 149.0455, 293.0874, 331.1756, 463.2176, 464.2212 |
| 2 | Monoterpene-diol pentosyl-hexoside-2 | 59.0145, 71.0143, 89.0243, 101.0246, 113.0244, 149.0451, 293.0874,331.1759, 463.2178, 464.2211 |
| 3 | Monoterpene-diol pentosyl-hexoside-3 | 59.0144, 71.0144, 89.0244, 101.0246, 113.0243, 149.0456, 293.0874, 463.2179, 464.2213, 465.2338 |
| 4 | Monoterpene-diol pentosyl-hexoside-4 | 59.0144, 71.0143, 89.0242, 101.0244, 113.0241, 149.0452, 293.0874,331.1755, 463.2179, 464.2212 |
| 5 | Monoterpene-diol pentosyl-hexoside-5 | 59.0143, 71.0144, 89.0243, 101.0245, 113.0243, 293.0874, 331.1759, 463.2178, 464.2209 |
| 6 | Monoterpene-diol pentosyl-hexoside-6 | 59.0145, 71.0143, 89.0243, 101.0246, 113.0244, 149.0451, 293.0874, 331.1759, 463.2178, 464.2211 |
| 7 | Monoterpene-diol pentosyl-hexoside-7 | 59.0144, 71.0144, 89.0244, 101.0246, 113.0243, 149.0456, 293.0874, 331.1759, 463.2179, 464.2213 |
| 8 | Monoterpene-diol pentosyl-hexoside-8 | 59.0143, 71.0144, 89.0243, 101.0245, 113.0243, 293.0874, 331.1759, 463.2178, 464.2209 |
| 9 | Monoterpene-diol hexosyl-pentosyl-hexoside-1 | 89.0235, 101.0243, 242.9976, 161.0441, 331.1753, 463.2174, 464.2207, 465.2232 |
| 10 | Monoterpene-diol hexosyl-pentosyl-hexoside-2 | 89.0242, 101.0247, 129.9759, 161.0465, 331.176, 332.1798, 463.2182, 464.2218, 465.227 |
| 11 | Monoterpene-diol hexosyl-pentosyl-hexoside-3 | 89.0243, 101.0250, 113.0243, 129.9756, 161.0455, 331.1755, 332.1781, 463.2174, 464.2207, 465.2233 |
| 12 | Dihydro-monoterpene-diol pentosyl-hexoside | 59.0145, 61.9889, 71.0144, 89.0243, 101.0246, 119.0346, 131.0351, 149.0454, 191.0564, 465.2328 |
| 13 | Monoterpenol pentosyl-hexoside-1 | 59.0146, 71.0148, 89.0244, 101.0241, 113.0242, 131.0351, 161.0458, 293.0875, 315.1803 |
| 14 | Monoterpenol pentosyl-hexoside-2 | 59.0145, 71.0144, 89.0242, 101.0244, 113.0243, 131.0350, 161.0455, 293.0875, 315.1804 |
| 15 | Monoterpenol pentosyl-hexoside-3 | 71.0142, 89.0228, 101.0251, 113.0253, 131.0355, 161.0461, 174.9557, 293.0875, 315.1806, 339.8081 |
| 16 | Monoterpenol pentosyl-hexoside-4 | 59.0144, 71.0143, 89.0242, 101.0245, 113.0245, 131.0347, 161.0456, 293.0875, 315.1808 |
| 17 | Monoterpenol pentosyl-hexoside-5 | 59.0144, 71.0144, 89.0243, 101.0245, 113.0243, 131.0351, 161.0454, 293.0875, 315.1806 |
| 18 | Monoterpenol pentosyl-hexoside-6 | 59.0144, 71.0144, 89.0244, 101.0246, 113.0244, 131.0351, 161.0453, 293.0875, 315.1806 |
| 19 | Monoterpenol pentosyl-hexoside-7 | 59.0144, 71.0143, 89.0244, 101.0245, 113.0243, 131.0351, 293.0875, 315.1806 |
| 20 | Monoterpenol hexosyl-pentosyl-hexoside-1 | 89.0242, 101.0239, 146.9645, 248.9596, 315.1806, 384.9264, 447.2228, 448.2265 |
| 21 | Monoterpenol hexosyl-pentosyl-hexoside-2 | 89.0246, 101.0248, 146.9664, 179.0573, 248.9599, 315.1806, 384.9367, 447.2223, 448.2257, 477.2335 |
| 22 | Monoterpenol hexosyl-pentosyl-hexoside-3 | 89.0245, 101.0256, 179.0576, 248.9598, 315.1816, 384.9345, 447.2222, 448.2286, 477.2347 |
| 23 | Monoterpenol rhamnosyl-hexoside | 59.0145, 61.989, 71.0144, 89.0244, 101.0246, 103.0403, 119.0348, 163.0612, 205.0717, 461.2385 |
| 24 | Geranic acid hexosyl-hexoside | 61.9888, 71.0145, 73.0299, 101.0244, 129.9758, 161.0454, 329.1599, 491.2125, 492.2153, 493.219 |
| 25 | Geranic acid rhamnosyl-hexoside | 59.0144, 61.9888, 71.0143, 89.0244, 101.0246, 103.0403, 119.0349, 163.0612, 167.1075, 307.1034, 475.2168 |
| 26 | Geranic acid pentosyl-hexoside-1 | 59.0142, 71.0144, 89.0243, 101.0247, 113.0245, 125.0234, 131.0351, 149.0453, 167.1074, 191.0561, 293.0875 |
| 27 | Geranic acid pentosyl-hexoside-2 | 59.0144, 71.0143, 89.0243, 101.0245, 113.0243, 125.0241, 131.0349, 149.0453, 167.1073, 191.0561, 293.0875 |
| 28 | Geranic acid pentosyl-hexoside-3 | 59.0143, 71.0143, 89.0244, 101.0245, 113.0244, 125.0241, 131.0351, 149.0454, 167.1075, 191.0562, 293.0880 |
| 29 | Geranic acid pentosyl-hexoside-4 | 59.0144, 71.0143, 89.0243, 101.0245, 113.0243, 125.0241, 131.0349, 149.0453, 167.1073, 191.0561, 293.0875 |
| 30 | Citronellol pentosyl-hexoside-1 | 59.0140, 71.0141, 85.0297, 101.0247, 113.0246, 161.0451, 317.1961, 318.1989, 449.2386, 450.2418 |
| 31 | Citronellol pentosyl-hexoside-2 | 59.0139, 71.0143, 89.0243, 101.0246, 113.0237, 161.0446, 317.1963, 318.1999, 449.2378, 450.2417 |
| 32 | Vomifoliol hexoside-1 | 59.0144, 71.0143, 89.0243, 101.0244, 113.0242, 152.0839, 153.0921, 161.0453, 179.0554, 205.1231, 223.1332, 385.1854 |
| 33 | Vomifoliol hexoside-2 | 59.0146, 71.0144, 89.0242, 101.0246, 113.0242, 152.0841, 153.0922, 161.0454, 179.0554, 205.123, 223.1331, 385.1856 |
| 34 | Vomifoliol pentosyl-hexoside | 71.0143, 89.0243, 101.0239, 125.0243, 131.0345, 149.0458, 153.0921, 205.1229, 233.0659, 293.0877 |
| 35 | Vomifoliol rhamnosyl-hexoside | 59.0146, 73.0296, 89.0245, 101.025, 103.0405, 112.9852, 119.0348, 163.0611, 205.0718, 307.1044, 325.1126 |
| 36 | 3-Oxo-𝛼-ionol/3-hydroxy-β-damascenone pentosyl-hexoside-1 | 59.0141, 71.0137, 89.0243, 101.0250, 113.024, 131.0350, 149.0455, 150.0487, 191.0555, 311.0974 |
| 37 | 3-Oxo-𝛼-ionol/3-hydroxy-β-damascenone pentosyl-hexoside-2 | 59.0144, 71.0144, 89.0244, 101.0245, 113.0242, 131.0348, 149.0454, 150.0489, 191.0557, 311.0984 |
| 38 | 3-Oxo-𝛼-ionol/3-hydroxy-*β*-damascenone rhamnosyl-hexoside | 59.0144, 73.0297, 89.0243, 101.0246, 103.0403, 119.0349, 143.0351, 163.0609, 205.0715, 265.093, 307.1044, 325.1126 |
| 39 | Benzyl alcohol pentosyl-hexside-1 | 59.0140, 71.0143, 89.0242, 101.0241, 113.0239, 161.0452, 269.1024, 270.1049, 293.0871 |
| 40 | Benzyl alcohol pentosyl-hexside-2 | 59.0145, 71.0143, 73.0297, 89.0242, 101.0246, 113.0243, 161.0455, 269.1024, 270.1059, 293.0871 |
| 41 | Benzyl alcohol pentosyl-hexside-3 | 59.0147, 71.0144, 73.0297, 89.0239, 101.0241, 113.0244, 161.0456, 269.1031, 270.1084, 293.0871 |
| 42 | Benzyl alcohol hexoside | 59.0144, 71.0144, 89.0245, 101.0246, 113.0245, 119.0347, 143.0348, 161.0456, 177.0558, 179.0559, 195.0659 |
| 43 | *β*-Phenylethanol pentosyl-hexoside | 59.0143, 71.0143, 89.0243, 99.0089, 101.0245, 131.0351, 149.0452, 161.0452, 191.0562, 283.1168 |
| 44 | *β*-Phenylethanol rhamnosyl-hexoside | 59.0142, 89.0247, 101.0246, 103.0403, 119.0347, 163.0616, 205.072, 265.0929, 325.1119,429.1767 |
| 45 | Methyl-salicylate rhamnosyl-hexoside | 59.0146, 71.0146, 89.0242, 101.0247, 103.0403, 113.0245, 137.0241, 143.035, 151.0402, 307.1049, 459.1501 |
| 46 | 3-Hexen-1-ol hexosyl-hexoside | 59.0146, 73.0298, 101.0245, 187.0974, 199.1343, 201.1132, 243.1242, 244.1247, 261.1340, 262.1381 |
| 47 | 1-Nonanol pentosyl-hexoside | 59.0142, 71.0143, 89.0244, 101.0243, 119.0346, 131.0350, 149.0452, 191.0562, 293.0870 |
| 48 | 1-Hexanol pentosyl-hexoside-1 | 59.0143, 89.0254, 101.0239, 113.0231, 131.0348, 161.0453, 263.1494, 264.1529, 395.1917, 396.1936 |
| 49 | 1-Hexanol pentosyl-hexoside-2 | 59.0144, 89.0242, 101.0244, 113.0237, 131.0358, 161.0447, 263.1500, 264.152, 395.1911, 396.195 |
| 50 | 1-Hexanol rhamnosyl-hexoside | 71.0144, 73.0295, 89.0241, 101.0247, 113.0241, 161.0454, 263.1496, 264.1525, 409.2069, 410.2104 |
| 51 | 3-Hexen-1-ol hexosyl-pentosyl-hexoside | 59.0146, 71.0145, 89.0238, 101.0250, 113.024, 119.0354, 129.975, 143.0347, 131.0457, 167.1071, 221.0658, 323.0967 |
| 52 | Isopropyl-alcohol pentosyl-hexoside | 59.0146, 71.0144, 73.0294, 83.0141, 85.0292, 89.0244, 99.0088, 101.0249, 113.024, 143.0346, 221.1026 |
| 53 | Furaneol pentosyl-hexoside | 59.0146, 61.9887, 71.0142, 73.0295, 85.0286, 89.0238, 99.009, 101.0249, 113.0239, 143.0329, 289.0919 |
| 54 | 2-Butanol pentosyl-hexoside | 59.0144, 71.0144, 73.0297, 85.0298, 89.0246, 101.0243, 113.0246, 131.0345, 161.0456, 235.1182 |
| 55 | 3-Methyl-1-butanol pentosyl-hexoside-1 | 59.0142, 71.0143, 73.0296, 89.024, 101.0242, 113.0239, 161.0451, 249.1345, 381.1755, 382.1785 |
| 56 | 3-Methyl-1-butanol pentosyl-hexoside-2 | 59.0147, 71.0141, 73.0292, 89.0246, 101.024, 113.0243, 161.0453, 249.1341, 381.1756, 382.1797 |
| 57 | 3-Methyl-1-butanol pentosyl-hexoside-3 | 59.0144, 71.0142, 73.0296, 89.0243, 101.0245, 113.0242, 161.0457, 249.1345 |
| 58 | 3-Methyl-2-buten-1-ol pentosyl-hexoside | 59.0141, 71.0141, 89.0243, 99.0091, 101.0244, 113.0245, 119.0346, 131.0346, 143.0342, 149.0455,249.1345 |
| 59 | 1,10-decanediol pentosyl-hexoside-1 | 61.9888, 71.0143, 101.0245, 161.0452, 335.2069, 336.2103, 467.2486, 468.2519, 469.2543 |
| 60 | 1,10-decanediol pentosyl-hexoside-2 | 61.9889, 71.0142, 101.0248, 161.0458, 335.2071, 336.2099, 467.2491, 468.2526, 469.2547 |

**The number (No.) correspond to compounds in* ***Table1****,* ***Supplementary Table 3****,* ***Figures 1****,* ***5****,* ***6****.*
