# Supplementary material for: Comparative Analysis of Glycosidic Aroma Compound Profiling in Three Vitis vinifera Varieties by Using Ultra-High-Performance Liquid Chromatography Quadrupole-Time-of-Flight Mass Spectrometry
Source: Front Plant Sci. 2021 Jun 24;12:694979. doi: 10.3389/fpls.2021.694979 (PMC8264444; doi:10.3389/fpls.2021.694979)
Supplement: Supplementary file 3 [file Table_3.docx]

**Supplementary Table 3** Relative concentrations of glycosidic aroma compounds in three grape varieties (two clones of each) over four ripening stages (µg internal standard/ g.fw)^1^.

| **No.**^2^ | M1^3^ | | | | M2 | | | | R1 | | | | R2 | | | | C1 | | | | C2 | | | |
| --- | --- | --- | --- | --- | --- | --- | --- | --- | --- | --- | --- | --- | --- | --- | --- | --- | --- | --- | --- | --- | --- | --- | --- | --- |
|  | 1^4^ | 2^4^ | 3^4^ | 4^4^ | 1 | 2 | 3 | 4 | 1 | 2 | 3 | 4 | 1 | 2 | 3 | 4 | 1 | 2 | 3 | 4 | 1 | 2 | 3 | 4 |
| **Monoterpenes** | | | | | | | | | | | | | | | | | | | | | | | |  |
| 1 | 0.81 | 0.80^e^ | 0.49^hi^ | 0.44^i^ | 0.92^d^ | 0.61^fg^ | 0.53^gh^ | 0.53^h^ | 1.56^a^ | 1.31^bc^ | 1.26^c^ | 0.69^f^ | 1.49^a^ | 1.49^a^ | 1.54^a^ | 1.38^b^ | nd | nd | nd | nd | nd | nd | nd | nd |
| 2 | 0.24^cd^ | 0.29^bc^ | 0.30^bc^ | 0.34^ab^ | 0.21^d^ | 0.31^bc^ | 0.25^cd^ | 0.42^a^ | nd | nd | nd | nd | nd | nd | nd | nd | nd | nd | nd | nd | nd | nd | nd | nd |
| 3 | 0.35^c^ | 0.44^b^ | 0.44^b^ | 0.54^a^ | 0.25^de^ | 0.37^bc^ | 0.32^cd^ | 0.59^a^ | 0.13^fg^ | 0.13^fg^ | 0.21^ef^ | 0.20^ef^ | 0.10^g^ | 0.14^fg^ | 0.28^cde^ | 0.33^cd^ | nd | nd | nd | nd | nd | nd | nd | nd |
| 4 | 6.79^a^ | 5.81^b^ | 3.79^ef^ | 3.97^de^ | 5.18^c^ | 4.17^d^ | 3.67^f^ | 3.83^ef^ | 1.49^g^ | 1.51 | 1^ij^ | 0.57^l^ | 1.22^hi^ | 1.37^gh^ | 0.95^jk^ | 0.75^kl^ | nd | nd | nd | nd | nd | nd | nd | nd |
| 6 | 0.79^b^ | 0.78^b^ | 0.62^bc^ | 0.67^bc^ | 0.73^b^ | 0.51^c^ | 1.14^a^ | 0.50^c^ | nd | nd | nd | nd | nd | nd | nd | nd | nd | nd | nd | nd | nd | nd | nd | nd |
| 7 | 2.61^a^ | 2.62^a^ | 1.69 | 1.54^c^ | 2.39^ab^ | 1.81^bc^ | 2.17^abc^ | 1.70^c^ | 0.16^d^ | 0.16^d^ | 0.12^d^ | 0.08^d^ | 0.14^d^ | 0.13^d^ | 0.13^d^ | 0.12^d^ | nd | nd | nd | nd | nd | nd | nd | nd |
| 8 | 7.46^a^ | 7.05^a^ | 4.57^c^ | 5.44^b^ | 6.89^a^ | 5.57 | 3.70^d^ | 5.17^bc^ | 2.25^e^ | 2.22^e^ | 1.58^efg^ | 0.98^g^ | 1.82^ef^ | 1.9^ef^ | 1.56^efg^ | 1.29^fg^ | nd | nd | nd | nd | nd | nd | nd | nd |
| 9 | 0.16^a^ | 0.16^a^ | 0.12^b^ | 0.10^c^ | 0.09^d^ | 0.12^b^ | 0.08^e^ | 0.11^b^ | nd | nd | nd | nd | nd | nd | nd | nd | nd | nd | nd | nd | nd | nd | nd | nd |
| 10 | 0.14^b^ | 0.19^a^ | 0.13^b^ | 0.14^b^ | 0.11^d^ | 0.14^b^ | 0.12^c^ | 0.14^b^ | nd | nd | nd | nd | nd | nd | nd | nd | nd | nd | nd | nd | nd | nd | nd | nd |
| 11 | 0.43^b^ | 0.51^a^ | 0.38^c^ | 0.37^c^ | 0.30^d^ | 0.39^c^ | 0.31^d^ | 0.37^c^ | nd | nd | nd | nd | nd | nd | nd | nd | nd | nd | nd | nd | nd | nd | nd | nd |
| 12 | 5.28^a^ | 3.77^bc^ | 3.90^b^ | 3.48^d^ | 5.09^a^ | 3.52^d^ | 3.93^b^ | 2.85^e^ | 0.49^fg^ | 0.53^f^ | 0.4^fg^ | 0.26^gh^ | 0.53^f^ | 0.45^fg^ | 0.57^f^ | 0.56^f^ | nd | nd | nd | nd | nd | nd | nd | nd |
| 13 | 0.30^d^ | 0.43^a^ | 0.22^e^ | 0.36^b^ | 0.45^a^ | 0.34^bc^ | 0.31^cd^ | 0.32^cd^ | 0.16^f^ | 0.13^fg^ | 0.1^ghi^ | 0.07^i^ | 0.15^f^ | 0.10^hi^ | 0.13^fgh^ | 0.11^gh^ | nd | nd | nd | nd | nd | nd | nd | nd |
| 14 | 0.69^b^ | 0.86^a^ | 0.36^e^ | 0.51^c^ | 0.73^b^ | 0.51^c^ | 0.45^d^ | 0.44^d^ | 0.35^e^ | 0.27^gh^ | 0.23^h^ | 0.15^i^ | 0.32^efg^ | 0.27^gh^ | 0.33^ef^ | 0.28^fgh^ | nd | nd | nd | nd | nd | nd | nd | nd |
| 15 | 0.29^de^ | 0.14^efgh^ | 0.48^c^ | 2.25^a^ | 0.10^fgh^ | 0.52^c^ | 0.23^def^ | 0.80^b^ | 0.05^gh^ | 0.05^gh^ | 0.05^gh^ | 0.25^def^ | 0.02^h^ | 0.03^h^ | 0.19^efg^ | 0.38^cd^ | nd | nd | nd | nd | nd | nd | nd | nd |
| 16 | 0.58^fg^ | 1.18^f^ | 3.29^c^ | 8.94^a^ | 0.51^g^ | 2.62^d^ | 1.90^e^ | 4.11^b^ | 0.03^g^ | 0.05^g^ | 0.04^g^ | 0.11^g^ | 0.02^g^ | 0.02^g^ | 0.07^g^ | 0.13^g^ | nd | nd | nd | nd | nd | nd | nd | nd |
| 17 | 0.66^f^ | 1.21^e^ | 2.83^c^ | 5.00^a^ | 0.52^fg^ | 2.52^c^ | 1.89^d^ | 3.59^b^ | 0.11^fg^ | 0.12^fg^ | 0.17^fg^ | 0.14^fg^ | 0.07^fg^ | 0.07^fg^ | 0.18^fg^ | 0.25^fg^ | 0.03^g^ | 0.03^g^ | 0.03^g^ | 0.05^fg^ | 0.02^g^ | 0.02^g^ | 0.04^g^ | 0.03^g^ |
| 18 | 3.33^d^ | 2.93^d^ | 4.27^bc^ | 7.89^a^ | 3.03^d^ | 4.01^c^ | 3.26^d^ | 4.59^b^ | 0.11^e^ | 0.14^e^ | 0.1^e^ | 0.08^e^ | 0.11^e^ | 0.08^e^ | 0.09^e^ | 0.08^e^ | 0.04^e^ | 0.04^e^ | 0.03^e^ | 0.05^e^ | 0.04^e^ | 0.03^e^ | 0.03^e^ | 0.03^e^ |
| 19 | 0.59^e^ | 1.36^d^ | 3.01^b^ | 3.80^a^ | 0.55^e^ | 2.38^c^ | 2.27^c^ | 2.98^b^ | 0.19^ef^ | 0.21^ef^ | 0.21^ef^ | 0.14^ef^ | 0.15^ef^ | 0.14^ef^ | 0.22^ef^ | 0.22^ef^ | 0.06^f^ | 0.06^f^ | 0.04^f^ | 0.06^f^ | 0.05^f^ | 0.04^f^ | 0.06^f^ | 0.05^f^ |
| 20 | 0.12^de^ | 0.18^a^ | 0.10^f^ | 0.13^d^ | 0.16^b^ | 0.14^c^ | 0.12^e^ | 0.13^d^ | nd | nd | nd | nd | nd | nd | nd | nd | nd | nd | nd | nd | nd | nd | nd | nd |
| 25 | 0.29^f^ | 0.47^e^ | 0.71^d^ | 2.07^a^ | 0.41^ef^ | 0.9^c^ | 0.54^de^ | 1.34^b^ | 0.08^g^ | 0.06^g^ | 0.06^g^ | 0.05^g^ | 0.05^g^ | 0.05^g^ | 0.04^g^ | 0.04^g^ | nd | nd | nd | nd | nd | nd | nd | nd |
| 26 | 5.20^a^ | 2.80^c^ | 1.77^e^ | 2.52^cd^ | 5.61^a^ | 3.91^b^ | 1.93^de^ | 3.97^b^ | 0.19^f^ | 0.16^f^ | 0.13^f^ | 0.17^f^ | 0.16^f^ | 0.15^f^ | 0.13^f^ | 0.14^f^ | 0.09^f^ | 0.08^f^ | 0.07^f^ | 0.10^f^ | 0.09^f^ | 0.06^f^ | 0.08^f^ | 0.08^f^ |
| 27 | 0.89^d^ | 0.96^cd^ | 1.39^b^ | 1.63^a^ | 0.97^d^ | 1.42^b^ | 1.11^c^ | 1.68^a^ | 0.09^e^ | 0.07^e^ | 0.08^e^ | 0.06^e^ | 0.06^e^ | 0.06^e^ | 0.06^e^ | 0.07^e^ | 0.03^e^ | 0.03^e^ | 0.03^e^ | 0.04^e^ | 0.03^e^ | 0.03^e^ | 0.04^e^ | 0.04^e^ |
| 28 | 0.44^e^ | 0.42^e^ | 0.54^d^ | 1.05^a^ | 0.61^d^ | 0.74^c^ | 0.62^d^ | 0.94^b^ | 0.08^f^ | 0.10^f^ | 0.07^f^ | 0.05^f^ | 0.06^f^ | 0.04^f^ | 0.06^f^ | 0.07^f^ | 0.02^f^ | 0.02^f^ | 0.02^f^ | 0.02^f^ | 0.02^f^ | 0.01^f^ | 0.02^f^ | 0.01^f^ |
| 29 | 1.43^a^ | 1.08^c^ | 1.21^b^ | 0.95^d^ | 1.30^b^ | 0.97^d^ | 0.95^d^ | 1.02^cd^ | 0.17^e^ | 0.11^ef^ | 0.12^ef^ | 0.09^ef^ | 0.15^ef^ | 0.14^ef^ | 0.13^ef^ | 0.11^ef^ | 0.06^f^ | 0.06^f^ | 0.05^f^ | 0.04^f^ | 0.07^ef^ | 0.04^f^ | 0.05^f^ | 0.05^f^ |
| 30 | 0.27^bc^ | 0.22^e^ | 0.19^f^ | 0.52^a^ | 0.29^b^ | 0.25^cd^ | 0.18^f^ | 0.24^de^ | nd | nd | nd | nd | nd | nd | nd | nd | nd | nd | nd | nd | nd | nd | nd | nd |
| 31 | 0.09^b^ | 0.08^bc^ | 0.07^de^ | 0.14^a^ | 0.07^ef^ | 0.08^cd^ | 0.06^f^ | 0.07^f^ | nd | nd | nd | nd | nd | nd | nd | nd | nd | nd | nd | nd | nd | nd | nd | nd |
| sum | 40.23^ab^ | 36.77^b^ | 36.87^b^ | 54.79^a^ | 37.47^ab^ | 38.85^b^ | 32.04^b^ | 42.45^ab^ | 7.68^c^ | 7.36^c^ | 5.92^c^ | 4.13^c^ | 6.63^c^ | 6.63 | 6.66^c^ | 6.31^c^ | 0.32^c^ | 0.32^c^ | 0.26^c^ | 0.35^c^ | 0.33^c^ | 0.23^c^ | 0.31^c^ | 0.29^c^ |
| **Norisoprenoids** | | |  |  |  |  |  |  |  |  |  |  |  |  |  |  |  |  |  |  |  |  |  |  |
| 32 | 0.50^hi^ | 0.55^h^ | 0.39^j^ | 0.37^j^ | 0.43^ij^ | 0.38^j^ | 0.37^j^ | 0.36^j^ | 1.20^a^ | 1.02^bc^ | 0.97^cd^ | 0.89^de^ | 0.77^fg^ | 1.01^bc^ | 0.69^g^ | 0.74^fg^ | 1.00^bc^ | 1.04^bc^ | 1.08^b^ | 1.16^a^ | 0.76^fg^ | 0.80^ef^ | 0.78^fg^ | 0.75^fg^ |
| 33 | 0.62^ij^ | 0.89^gh^ | 0.68^ij^ | 0.69^ij^ | 0.61^j^ | 0.72^ij^ | 0.72^ij^ | 0.78^hi^ | 2.23^a^ | 1.73^cd^ | 2.04^b^ | 2.29^a^ | 1.57^e^ | 1.83^c^ | 1.65^de^ | 2.07^b^ | 1.23^f^ | 1.50^e^ | 1.63^de^ | 2.24^a^ | 1.00^g^ | 1.18^f^ | 1.50^e^ | 1.59^de^ |
| 34 | 0.21^g^ | 0.18^gh^ | 0.18^gh^ | 0.13^ij^ | 0.15^hij^ | 0.14 | 0.16^hi^ | 0.12^j^ | 0.61^a^ | 0.52^b^ | 0.46^c^ | 0.45^cd^ | 0.47^c^ | 0.57^a^ | 0.44^cde^ | 0.46^cd^ | 0.29^f^ | 0.44^cde^ | 0.41^de^ | 0.47^c^ | 0.28^f^ | 0.30^f^ | 0.40^e^ | 0.44^cde^ |
| 38 | nd | nd | nd | nd | nd | nd | nd | nd | 0.08^hi^ | 0.07^hi^ | 0.11^g^ | 0.22^d^ | 0.07^i^ | 0.1^gh^ | 0.14^f^ | 0.18^e^ | 0.19^e^ | 0.34^c^ | 0.38^b^ | 0.52^a^ | 0.15^f^ | 0.19^e^ | 0.34^c^ | 0.33^c^ |
| sum | 1.34^gh^ | 1.61^fgh^ | 1.25^h^ | 1.19^h^ | 1.19^h^ | 1.23^h^ | 1.25^h^ | 1.25^h^ | 4.12^ab^ | 3.34^abcd^ | 3.59^abcd^ | 3.85^abc^ | 2.88^cde^ | 3.50^abcd^ | 2.93^cde^ | 3.45^bcd^ | 2.71^cdef^ | 3.32^bcd^ | 3.50^abcd^ | 4.39^a^ | 2.19^efgh^ | 2.48^defg^ | 3.02^cde^ | 3.11^cde^ |
| **Benzenoids** | | |  |  |  |  |  |  |  |  |  |  |  |  |  |  |  |  |  |  |  |  |  |  |
| 41 | 1.17^fg^ | 1.39^f^ | 1.04^fg^ | 1.17^fg^ | 0.90^g^ | 1.1^fg^ | 0.99^fg^ | 1.1^fg^ | 3.48^bcd^ | 3.23^de^ | 2.82^e^ | 3.09^de^ | 3.06^e^ | 3.38^cd^ | 3.14^e^ | 3.07^de^ | 3.23^d^ | 3.45^bcd^ | 3.48^bcd^ | 3.82^ab^ | 4.12^a^ | 3.33^d^ | 3.91^a^ | 3.82^abc^ |
| 42 | 0.42^f^ | 0.31^ghij^ | 0.31^ghij^ | 0.24^ij^ | 0.39^fg^ | 0.28^hij^ | 0.27^hij^ | 0.26^hij^ | 1.60^a^ | 1.44^b^ | 1.19^c^ | 0.88^d^ | 1.17^c^ | 1.34^b^ | 0.93^d^ | 0.93^d^ | 0.20^j^ | 0.44^ef^ | 0.54^e^ | 0.45^ef^ | 0.43^f^ | 0.41^fg^ | 0.37^fgh^ | 0.32^ghi^ |
| 43 | 0.72^kl^ | 0.73^kl^ | 0.72^kl^ | 0.72^kl^ | 0.58^l^ | 0.72^l^ | 0.79^jk^ | 0.61^l^ | 1.13^fgh^ | 0.94^ij^ | 0.94^ij^ | 1.24^defg^ | 1.07^hi^ | 1.15^fgh^ | 1.25^defg^ | 1.31^cde^ | 1.08^ghi^ | 1.35^cde^ | 1.22^efgh^ | 1.38^bcd^ | 1.48^abc^ | 1.29^def^ | 1.53^ab^ | 1.56^a^ |
| 44 | 0.39^a^ | 0.35^b^ | 0.27^de^ | 0.35^b^ | 0.35^b^ | 0.3^c^ | 0.29^cd^ | 0.26^e^ | 0.15^g^ | 0.12^hi^ | 0.13^ghi^ | 0.25^e^ | 0.13 | 0.14^ghi^ | 0.14^ghi^ | 0.22^f^ | 0.07^j^ | 0.08^j^ | 0.08^j^ | 0.14^g^ | 0.14^g^ | 0.11^i^ | 0.13 | 0.14^gh^ |
| sum | 2.70^c^ | 2.78^c^ | 2.34^c^ | 2.47^c^ | 2.23^c^ | 2.4^c^ | 2.34^c^ | 2.23^c^ | 6.36^a^ | 5.73^ab^ | 5.08^ab^ | 5.46^ab^ | 5.43^ab^ | 6.02^ab^ | 5.46^ab^ | 5.54^ab^ | 4.59^b^ | 5.32^ab^ | 5.31^ab^ | 5.79^ab^ | 6.16^a^ | 5.15^ab^ | 5.95^ab^ | 5.85^ab^ |
| **C6/C9 compounds** | | |  |  |  |  |  |  |  |  |  |  |  |  |  |  |  |  |  |  |  |  |  |  |
| 46 | 0.53^a^ | 0.35^ef^ | 0.37^de^ | 0.29^gh^ | 0.31^gh^ | 0.28^hi^ | 0.26^i^ | 0.22^j^ | nd | nd | nd | nd | nd | nd | nd | nd | 0.41^bc^ | 0.44^b^ | 0.38^de^ | 0.38^de^ | 0.39^cd^ | 0.36^e^ | 0.31^g^ | 0.32^fg^ |
| 47 | 1.41^a^ | 1.05^c^ | 1.25^b^ | 1.20^b^ | 1.41^a^ | 1.03^c^ | 1.22^b^ | 0.98^c^ | 0.27^d^ | 0.27^d^ | 0.23^d^ | 0.16^e^ | 0.27^d^ | 0.26^d^ | 0.29^d^ | 0.29^d^ | nd | nd | nd | nd | nd | nd | nd | nd |
| 48 | nd | nd | nd | nd | nd | nd | nd | nd | 0.12^c^ | 0.12^c^ | 0.12^c^ | 0.19^a^ | 0.09^d^ | 0.13^c^ | 0.14^b^ | 0.14^b^ | 0.05^e^ | 0.09^d^ | 0.08^d^ | 0.14^b^ | 0.06^e^ | 0.06^e^ | 0.09^d^ | 0.12^c^ |
| 49 | nd | nd | nd | nd | nd | nd | nd | nd | 0.11^a^ | 0.10^ab^ | 0.10^b^ | 0.09^cd^ | 0.09^cd^ | 0.10^bc^ | 0.11^ab^ | 0.09^cd^ | 0.06^ef^ | 0.07^e^ | 0.07^e^ | 0.08^d^ | 0.06^ef^ | 0.06^fg^ | 0.06^fg^ | 0.06^g^ |
| 50 | nd | nd | nd | nd | nd | nd | nd | nd | 0.10^c^ | 0.09^de^ | 0.10^d^ | 0.11^b^ | 0.08^f^ | 0.09^e^ | 0.11^c^ | 0.12^a^ | nd | nd | nd | nd | nd | nd | nd | nd |
| sum | 1.94^a^ | 1.4^bc^ | 1.62^abc^ | 1.49^abc^ | 1.72^ab^ | 1.31^bc^ | 1.48^abc^ | 1.2^c^ | 0.52^d^ | 0.59^d^ | 0.53^d^ | 0.6^d^ | 0.51^d^ | 0.48^d^ | 0.47^d^ | 0.5^d^ | 0.6^d^ | 0.59^d^ | 0.55^d^ | 0.55^d^ | 0.54^d^ | 0.58^d^ | 0.65^d^ | 0.65^d^ |
| **Others** | | |  |  |  |  |  |  |  |  |  |  |  |  |  |  |  |  |  |  |  |  |  |  |
| 52 | nd | nd | nd | nd | nd | nd | nd | nd | 0.19^fg^ | 0.19^fg^ | 0.24^e^ | 0.32^b^ | 0.16^g^ | 0.18^fg^ | 0.32^bc^ | 0.40^a^ | 0.20^f^ | 0.25^de^ | 0.27^de^ | 0.42^a^ | 0.18^fg^ | 0.19^fg^ | 0.29^cd^ | 0.33^b^ |
| 53 | nd | nd | nd | nd | 0.23^a^ | 0.16^b^ | 0.16^b^ | 0.09^c^ | nd | nd | nd | nd | nd | nd | nd | nd | nd | nd | nd | nd | nd | nd | nd | nd |
| 54 | 0.96^b^ | 1.06^a^ | 0.60^e^ | 0.54^f^ | 1.06^a^ | 0.81^c^ | 0.73^d^ | 0.69^d^ | 0.27^j^ | 0.26^hij^ | 0.23^k^ | 0.34^g^ | 0.24^hijk^ | 0.24^k^ | 0.26^hij^ | 0.28^ghi^ | 0.26 | 0.2^jk^ | 0.2^jk^ | 0.28^gh^ | 0.18^k^ | 0.18^k^ | 0.24^hijk^ | 0.21^ijk^ |
| 55 | 0.27^fg^ | 0.27^fg^ | 0.25^g^ | 0.34^defg^ | 0.27^fg^ | 0.27^fg^ | 0.29^efg^ | 0.22^g^ | 0.99^ab^ | 0.68^c^ | 0.52^cdef^ | 0.71^c^ | 0.63^c^ | 0.66^c^ | 0.62^c^ | 0.69^c^ | 0.57^cd^ | 0.61^c^ | 0.59^cd^ | 0.72^bc^ | 0.56^de^ | 0.57^cde^ | 1.00^a^ | 1.10^a^ |
| 58 | 0.57^hi^ | 0.62^gh^ | 0.90^ab^ | 0.98^a^ | 0.50^i^ | 0.79^cde^ | 0.81^bcd^ | 0.89^ab^ | 0.61^h^ | 0.57^hi^ | 0.71^efg^ | 0.65^fgh^ | 0.57^hi^ | 0.72^defg^ | 0.74^cdef^ | 0.82^bc^ | 0.63^gh^ | 0.77^cde^ | 0.74^cdef^ | 0.81^bc^ | 0.66^fgh^ | 0.65^fgh^ | 0.75^cde^ | 0.75^cde^ |
| 59 | 0.78^a^ | 0.60^b^ | 0.46^d^ | 0.43^d^ | 0.81^a^ | 0.47^cd^ | 0.52^c^ | 0.28^e^ | nd | nd | nd | nd | nd | nd | nd | nd | nd | nd | nd | nd | nd | nd | nd | nd |
| 60 | 0.67^b^ | 0.55^c^ | 0.30^e^ | 0.38^d^ | 0.79^a^ | 0.42^d^ | 0.44^d^ | 0.23^f^ | nd | nd | nd | nd | nd | nd | nd | nd | nd | nd | nd | nd | nd | nd | nd | nd |
| sum | 3.24^ab^ | 3.10^abc^ | 2.50^cdef^ | 2.67^bcde^ | 3.65^a^ | 2.92^bcd^ | 2.96^bcd^ | 2.41^cdef^ | 2.06^efghi^ | 1.71^fghi^ | 1.7^fghi^ | 2.03^efghi^ | 1.6^hi^ | 1.80^fghi^ | 1.93^efghi^ | 2.20^defghi^ | 1.65^ghi^ | 1.83^fghi^ | 1.80^fghi^ | 2.24^defgh^ | 1.58^i^ | 1.60^hi^ | 2.27^defg^ | 2.39^cdefg^ |
| **TOTAL** | 49.45^b^ | 45.67^ab^ | 44.59^b^ | 62.62^a^ | 46.27^ab^ | 46.72^ab^ | 40.06^bc^ | 49.54^ab^ | 20.83^cd^ | 18.73^d^ | 16.84^d^ | 16.02^d^ | 17.07^d^ | 18.53^d^ | 17.63^d^ | 18.15^d^ | 9.78 ^d^ | 11.38^d^ | 11.4^d^ | 13.37^d^ | 10.77^d^ | 9.93^d^ | 12.02^d^ | 12.14 ^d^ |

*^1^ Per compound, mean values with different letters are significantly different at p ≤ 0.05; n = 4; 2 biological replicates × 2 analytical replicates.*

*^2^ M1, M2, R1, R2, C1 and C2 correspond to the clone 1 and clone2 of Muscat Blanc (M), Riesling (R) and Chardonnay (C), respectively.*

*^3^ The number (No.) correspond to compounds in* ***Table 1****,* ***Supplementary Table 2****,* ***Figures 1****,* ***5****,* ***6****.*

*^4^ 1,2,3,4 refer to E-L34, E-L35, E-L36, 3-L37. nd: not detected.*
