# Supplementary material for: Comparative Analysis of Glycosidic Aroma Compound Profiling in Three Vitis vinifera Varieties by Using Ultra-High-Performance Liquid Chromatography Quadrupole-Time-of-Flight Mass Spectrometry
Source: Front Plant Sci. 2021 Jun 24;12:694979. doi: 10.3389/fpls.2021.694979 (PMC8264444; doi:10.3389/fpls.2021.694979)
Supplement: Supplementary file 4 [file Table_4.docx]

**Supplementary Table 4** Some data about ripening of the three grape varieties (two clones of each).

|  | Stages | °Brix^1^ | pH^1^ |
| --- | --- | --- | --- |
| M1^2^ | E-L34 | 6.0 ± 0.0 | 2.47 ± 0.01 |
|  | E-L35 | 10.9 ± 0.0 | 2.98 ± 0.00 |
|  | E-L36 | 13.7 ± 0.1 | 3.18 ± 0.01 |
|  | E-L37 | 16.2 ± 0.1 | 3.22 ± 0.01 |
| M2 | E-L34 | 6.4 ± 0.0 | 2.47 ± 0.01 |
|  | E-L35 | 10.1 ± 0.0 | 2.88 ± 0.0 |
|  | E-L36 | 13.9 ± 0.1 | 2.98 ± 0.01 |
|  | E-L37 | 14.5 ± 0.0 | 3.30 ± 0.00 |
| R1 | E-L34 | 7.1 ± 0.0 | 2.68 ± 0.01 |
|  | E-L35 | 10.6 ± 0.0 | 2.74 ± 0.01 |
|  | E-L36 | 14.7 ± 0.1 | 2.97 ± 0.01 |
|  | E-L37 | 13.2 ± 0.0 | 3.31 ± 0.01 |
| R2 | E-L34 | 7.6 ± 0.05 | 2.69 ± 0.00 |
|  | E-L35 | 10.3 ± 0.0 | 2.62 ± 0.01 |
|  | E-L36 | 14.2 ± 0.10 | 3.20 ± 0.01 |
|  | E-L37 | 13.5 ± 0.0 | 3.35 ± 0.01 |
| C1 | E-L34 | 6.5 ± 0.1 | 2.83 ± 0.01 |
|  | E-L35 | 12.2 ± 0.1 | 2.93 ± 0.01 |
|  | E-L36 | 14.3 ± 0.1 | 2.91 ± 0.00 |
|  | E-L37 | 15.6 ± 0.0 | 3.57 ± 0.0 |
| C2 | E-L34 | 6.3 ± 0.0 | 2.88 ± 0.00 |
|  | E-L35 | 11.7 ± 0.0 | 2.86 ± 0.01 |
|  | E-L36 | 14.0 ± 0.1 | 3.24 ± 0.01 |
|  | E-L37 | 15.2 ± 0.1 | 3.26 ± 0.00 |

*^1^ Three analytical replicates were included.*

*^2^ M1, M2, R1, R2, C1 and C2 correspond to the clone 1 and clone2 of Muscat Blanc (M), Riesling (R) and Chardonnay (C), respectively.*
